# Systematic profiling of cancer‐fibroblast interactions reveals drug combinations in ovarian cancer

**DOI:** 10.1002/1878-0261.70051

**Published:** 2025-05-24

**Authors:** Greta Gudoityte, Osheen Sharma, Laura Leuenberger, Emelie Wallin, Josefin Fernebro, Päivi Östling, Rebecka Bergström, Johan Lindberg, Ulrika Joneborg, Olli Kallioniemi, Brinton Seashore‐Ludlow

**Affiliations:** ^1^ Science for Life Laboratory, Department of Oncology‐Pathology Karolinska Institutet Stockholm Sweden; ^2^ Department of Oncology and Pathology Karolinska Institutet Stockholm Sweden; ^3^ Department of Pelvic Cancer Karolinska University Hospital Stockholm Sweden; ^4^ Department of Molecular Epidemiology and Biostatistics Karolinska Institutet Stockholm Sweden; ^5^ Department of Women's and Children's Health Karolinska Institutet Stockholm Sweden; ^6^ Institute for Molecular Medicine Finland University of Helsinki Finland

**Keywords:** cancer‐associated fibroblast, drug screen, ovarian cancer, tumor microenvironment

## Abstract

Ovarian cancer (OC) is a leading cause of death of gynecological cancers in women. Poor patient response to treatment highlights the need to better understand how the tumor microenvironment affects OC progression. Growing evidence indicates the crucial role of non‐cancerous components, such as cancer‐associated fibroblasts, in establishing a complex network of cellular and molecular interactions, influencing cancer progression and response to treatment. Therefore, in this study, we sought to characterize the impact of fibroblasts on OC cell behavior and drug response. Using both direct and indirect cell co‐culture systems, we observed distinct changes in cancer cell proliferation, morphology, and secretome in the presence of fibroblasts. Furthermore, an imaging‐based high‐throughput drug screen of 528 oncology compounds revealed multiple drugs that showed altered efficacy in the co‐culture conditions, demonstrating the role of fibroblasts in driving cancer cell resistance to treatment. Most importantly, our data identified the two drug combinations of Birinapant or Vorinostat with Carboplatin as promising treatments, exploiting the altered cancer cell phenotype in co‐cultures. These findings were supported by the increased sensitivity of *ex vivo* cultures to these combinations.

## Graphical abstract

Fibroblasts, cells in the tumor environment, support ovarian cancer cell growth and alter morphology and drug response. We used fibroblast and cancer cell co‐culture models to test 528 drugs and discovered new drugs for combination treatment. We showed that adding Vorinostat or Birinapant to standard chemotherapy may improve drug response, suggesting new ways to treat ovarian cancer.

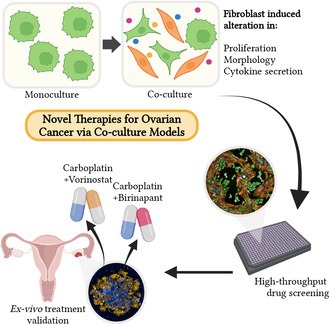

**Abbreviations**
ANAX1: annexin A1
AREG: amphiregulin
*BRCA1/2*
: breast cancer type ½ susceptibility protein
BSA: bovine serum albumin
CA‐125: cancer antigen 125 (mucin‐16)
CAFs: cancer‐associated fibroblasts
CD31: platelet and endothelial cell adhesion molecule
CD90: thy‐1 cell surface antigen
CK8/18: keratin 8/18
CM: conditioned media
CNAs: copy number alterations
CP: CellProfiler analyst
CPE: carboxypeptidase E
CTSV: cathepsin V
D‐CC: direct co‐culture
DMSO: dimethyl sulfoxide
ECM: extracellular matrix components
EDTA: ethylenediaminetetraacetic acid
EMT: epithelial‐mesenchymal transition
EpCam: epithelial cell adhesion molecule
ERBB4: erb‐b4 receptor tyrosine kinase
FBS: fetal bovine serum
FC: fold change
FIMM: Institute Molecular Medicine Finland
FOV: field of view
FSP‐1: fibroblast‐specific protein‐1
Gal‐1: galectin‐1
GPNMB: transmembrane glycoprotein NMB
HDAC: histone deacetylase
HGF: hepatocyte growth factor
HGSOC: high grade serous ovarian cancer
hK8: kallrikrein‐8
HRD: homologous recombination deficiency
I‐CC: indirect co‐culture
IC: IncuCyte^®^ software
IF: immunofluorescence
ITGB5: integrin beta‐5
JAK1: Janus kinase 1
KUH: Karolinska University Hospital
MEK: mitogen‐activated protein kinase kinase
MoA: mechanism of action
mTORC1/2: mammalian target of rapamycin complex 1/2
NF: non‐cancerous fibroblasts
NGI: national genomics infrastructure
NPX: Normalized Protein Expression
PARP: poly (ADP‐ribose) polymerase
PBS: phosphate‐buffered saline
PDFs: Patient‐derived fibroblasts
PDPN: podoplanin
PEGPH20: Pegylated recombinant human hyaluronidase
PFA: paraformaldehyde
PI3K: phosphoinositide 3‐kinase
RSPO3: R‐spondin
RT: room temperature
SD: standard deviation
sDSS: selective drug sensitivity score
SMAC: second mitochondrial‐derived activator of caspases
SMAD5: mothers against decapentaplegic homolog 5
SPARC: secreted protein acidic and rich in cysteine
ssGSEA: single sample gene set enrichment analysis
STAT: signal transducer and activator of transcription
STR: short‐tandem repeat
TFPI‐2: tissue factor pathway inhibitor 2
TGF‐β: transforming growth factor beta
TLR3: toll‐like receptor 3
TME: tumor microenvironment,
TMRM: tetramethylrhodamine, methyl ester, perchlorate
TNFRSF1: tumor necrosis factor receptor 1
*TP53*
: *tumor protein 53*
VEGFA: vascular endothelial growth factor A
VIM: vimentin
WFDC2: epididymis protein‐4
WISP‐1: WNT1‐inducible signaling pathway protein 1
XIAP: X‐linked inhibitor of apoptosis protein
α‐SMA: alpha‐smooth muscle actin α‐SMA

## Introduction

High grade serous ovarian cancer (HGSOC) is the most common and prevalent subtype of OC [1, 2]. It is characterized by high copy number alterations (CNAs) and few recurrent genetic mutations in genes associated with targeted therapies. Instead, 95% of patients harbor mutations in *TP53*, leading to impaired DNA repair, altered cell cycle regulation, and increased cell proliferation [3]. Currently, most patients undergo cytoreductive debulking surgery followed by platinum–taxol chemotherapy. While initial response to treatment is favorable for most patients, approximately 80% relapse within 5 years with an average recurrence time of 18 months [4]. Secondary disease management typically involves additional platinum‐based chemotherapy, but many patients develop resistance, leaving limited treatment options [5]. The improved understanding of the genetic alterations, such as *BRCA1/2* mutations and high prevalence of homologous recombination repair deficiencies in HGSOC has led to the use of poly (ADP‐ribose) polymerase (PARP) inhibitors and antiangiogenic drugs [6]. However, long‐term survival rates for these patients have not significantly improved [7], highlighting the need to explore HGSOC vulnerabilities beyond cancer cells and their genetic alterations.

Growing evidence suggests the importance of non‐cancerous components, such as fibroblasts, adipocytes, and immune cells, in shaping tumor progression and response to treatment [8]. Despite this, research in cancer therapeutics development has traditionally focused on intrinsic cancer cell vulnerabilities. While this approach has advanced our understanding of HGSOC and targetable disease mechanisms, it overlooks the complexity of the tumor and its environment, potentially missing opportunities for the development of novel treatment strategies [9]. Cancer‐associated fibroblasts (CAFs), a major component of the tumor microenvironment (TME), play a crucial role in cancer development and drug response. These cells secrete extracellular matrix components (ECM) (such as collagen, laminin etc.) and thus induce changes in the biomechanical properties of the tumor. This can then obstruct drug delivery to cancer cells. In addition, secretory molecules and exosomes from CAFs provide cancer cells with signals inhibiting apoptosis or leading to drug resistance through altered gene expression, ultimately causing increased cell survival [10, 11]. Therefore, recent efforts have been directed toward the development of targeted treatments disrupting the signals emanating from the TME and thereby enhancing tumor cell killing. These strategies include targeting fibroblast markers to eliminate CAFs, reversing the phenotype of CAFs, or modulating downstream effects of CAFs. However, this remains an elusive goal, and there is a need to understand how to leverage these vulnerabilities to improve OC treatment [10].

In this study, we aim to characterize the impact of fibroblasts on OC cell behavior. Given the lack of representative models to study the effects of the CAF, we have established an *in vitro* co‐culture system enabling the use of patient‐derived and normal fibroblasts. Using this system, we evaluated the direct and indirect effects of fibroblasts on cancer cell proliferation, morphology, and secretory profile. Further, we performed high‐throughput drug screening to identify drugs that sensitize cancer cells in a co‐culture setting. Our assay identified the histone deacetylase (HDAC) inhibitor, Vorinostat, and the second mitochondrial‐derived activator of caspases (SMAC) mimetic, Birinapant, as drugs with higher selective killing potential in co‐culture. The clinical relevance of these drugs was validated in *ex vivo* OC cultures, suggesting further studies to validate their potential use in combination with Carboplatin for patients with OC.

## Material and methods

### Study overview

**Fig. 1:**
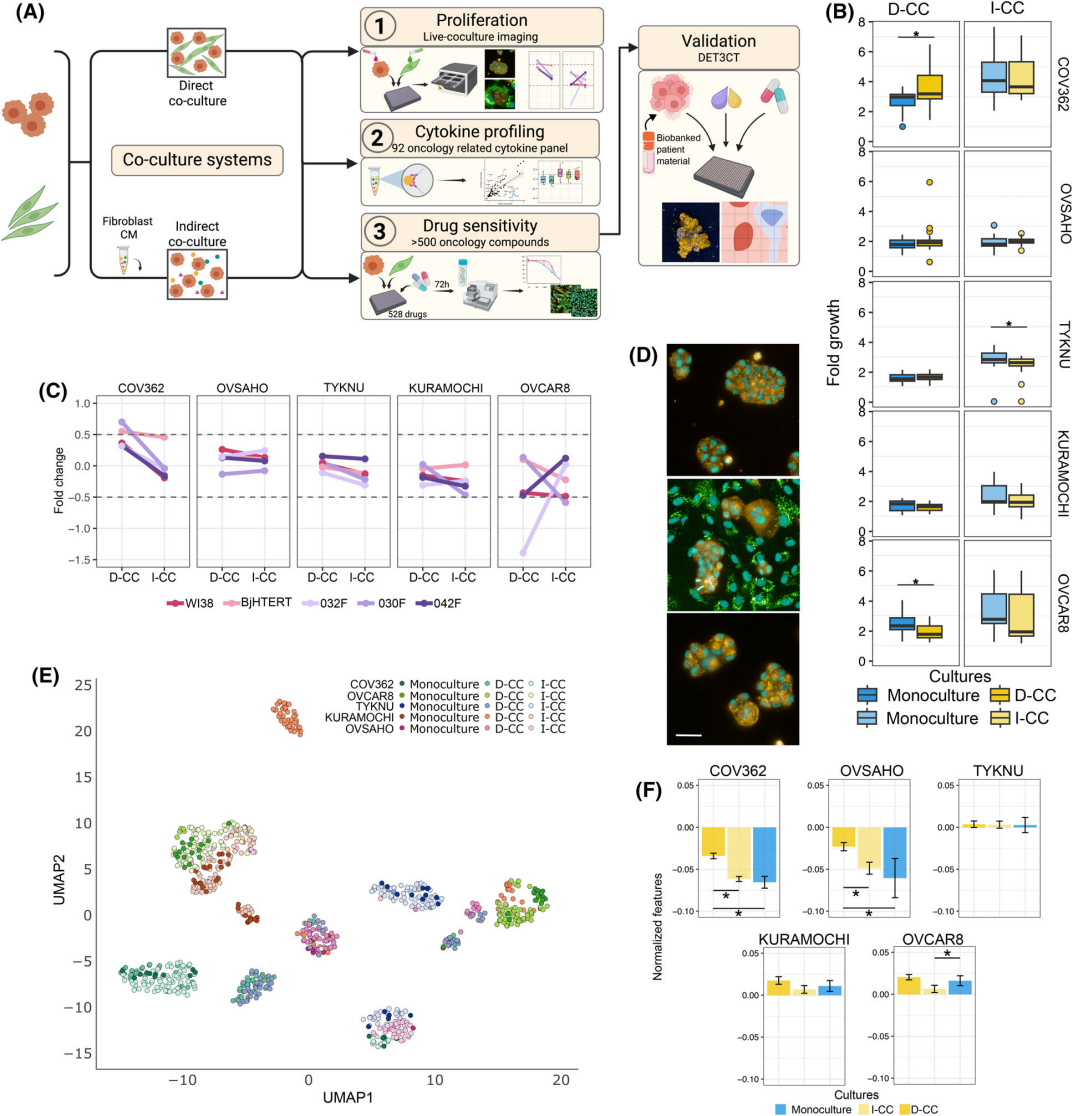
Cancer and fibroblast cell interactions alter cancer cell proliferation and morphology. (A) Schematic overview of the methods used in this study to characterize and study cancer and fibroblast interactions (Figure created with Biorender.com). (B) Fold change of cancer cell growth after 72 h in monoculture and co‐culture averaged for all fibroblast cultures at a 1 : 1 ratio. Data presented as mean ± SD (*n* = 8 for each condition), *P < 0.05 from Mann‐Whitney U test. (C) Comparison between direct co‐cultures (D‐CC) and indirect co‐cultures (I‐CC) fold change for cancer cells after 72 h in culture with the five different fibroblasts at a 1 : 1 ratio. (D) Representative images of OVSAHO cell morphology in monoculture (top), D‐CC (middle), I‐CC (bottom) panel, scale bar 200 μm (yellow is pre‐stained cancer cells, green is pre‐stained fibroblast cells, and turquoise is nuclear staining). (E) UMAP representation of the distribution of 318 morphological features extracted from the images of cancer cells under different culture conditions. Each cell line and culture condition are represented by the color scheme found in the figure legend. Each dot represents averaged cancer cell features per well, *n* = 4–8 replicates. (F) Comparison of normalized morphological features (*n* = 318) between monoculture and co‐culture conditions for each cancer cell line, averaged for all fibroblast co‐cultures (*n* = 4–8 for each condition). Data presented as mean of ± SD (*n* = 4–8 for each condition), *P < 0.05 from Mann–Whitney U test.

OC (*n* = 5) and fibroblast (*n* = 5) cell lines were used for this study. To assess changes induced by cell interactions, we designed a co‐culture system enabling us to capture the effects of cancer and fibroblast cell populations in live and fixed cell settings (Fig. 1A). To investigate contact‐dependent effects, cells were cultured in direct co‐culture (D‐CC), while for contact‐independent effects, cancer cells were grown in media supplemented with fibroblast conditioned media (CM), creating an indirect co‐culture (I‐CC). Co‐cultures and monocultures were used to determine changes in cancer cell proliferation, morphology, cytokine secretion profile, drug sensitivity, and further findings were validated using *ex vivo* spheroid cultures representing HGSOC patients.

### Cell culture

OC cell lines KURAMOCHI (RRID:CVCL_1345), OVCAR8 (RRID:CVCL_1629) and OVSAHO (RRID:CVCL_3114) were maintained in RPMI (BioWest, Sigma‐Aldrich, Burlington, MA, USA) medium; COV362 (RRID:CVCL_2420) (Sigma‐Aldrich, USA) and TYKNU (RRID:CVCL_1776) were cultured in DMEM (Sigma‐Aldrich) medium. All cancer cell line media were supplemented with 10% fetal bovine serum (FBS) (Gibco, Thermo Fisher Scientific, Waltham, MA, USA), 1× antibiotics (streptomycin/penicillin) (Gibco, Thermo Fisher Scientific) and 1X L‐glutamine (Gibco, Thermo Fisher Scientific). Skin fibroblast cell line BJHTERT (RRID:CVCL_6573) was maintained in DMEM supplemented with 14.5% FBS, 16.5% Medium 199 (Sigma‐Aldrich), and 1X antibiotics; lung fibroblast cell line WI38 (RRID:CVCL_0579) (VWR, USA) was cultured in MEM/EBSS (Gibco, Thermo Fisher Scientific) supplemented with 10% FBS, 1× antibiotics, and 1× non‐essential amino acids (Sigma‐Aldrich). Patient‐derived fibroblasts (PDFs) were cultured in DMEM media supplemented with 10% FBS, 1× antibiotics, 1× l‐glutamine, 5 μg·mL^−1^ insulin (Merk, Germany), 0.4 μg·mL^−1^ hydrocortisone (Sigma‐Aldrich), and 5 ng·mL^−1^ basic fibroblast growth factor (Peprotech, Thermo Fisher Scientific). All cells were maintained in a 5% CO_2_‐humidified incubator at 37 °C and split once confluency reached ~70%. Cancer cell lines and BJHTERT fibroblasts were obtained from the Institute Molecular Medicine Finland (FIMM) and provided by Dr. Astrid Muramägi. All commercial cell lines (Table S1) were validated by short‐tandem repeat (STR) sequencing (Eurofins, Luxembourg) in the past 3 years. Cells were regularly screened for mycoplasma contamination (Lonza, Switzerland) according to the manufacturer's protocol. All experiments were performed with mycoplasma‐free cells.

### Patient‐derived fibroblast model establishment

Patient‐derived fibroblasts were derived from OC patient tumor tissue material. Samples were obtained from Karolinska University Hospital (KUH) in Stockholm. The study was conducted under approved ethical permits (nr. 2016/1197–31/1, nr. 2018/2642–32 and nr. 2018/118–32) from the Swedish Ethical Review Authority (Etikprövningsmyndigheten). Patients signed informed consent prior to material collection. Samples were processed as described by Åkerlund et al. [12] using the gentleMACS tissue dissociation kit (Miltenyi, Germany). After tissue dissociation, cells were plated into cell culture flasks. Once the flask reached 70–80% confluency, differential trypsinization was performed. Specifically, after 3 min incubation with TrypLE (Invitrogen, Waltham, MA, USA), the dissociated cell fraction was collected, resuspended in fibroblast culture media, and spun down. Once consistent proliferation was achieved, these cells were expanded for biobanking and validation. Clinical information describing the characteristics of the patient material is provided in Table S1.

### Patient‐derived fibroblast mutation characterization

Snap‐frozen tumor tissue, blood, ascites, and PDF cell pellets were used to perform target sequencing. DNA was extracted with AllPrep DNA/RNA kit (Qiagen, Hilden, Germany) using the manufacturer's recommendations, concentration assessed with Qubit fluorometer (Invitrogen). Further material preparation, sequencing, and analysis was performed in National Genomics Infrastructure (NGI) as described previously [13]. Briefly, library preparation was done with Kapa DNA HyperPrep (Roche, Bazel, Switzerland) further target‐based sequencing of ProBio panel (v3) was performed using Illumina paired‐end sequencing on Nova Seq system (Illumina, San Diego, CA, USA). Subsequent data processing was conducted using the in‐house bioinformatics pipeline AutoSeq.

### Live‐cell co‐culture staining

Prior to plating for live‐cell imaging in co‐cultures, cells were labeled with PKH‐26 and PKH‐67 (Sigma‐Aldrich), following the manufacturer's recommendations. Dye concentrations were optimized for each cell line individually, ranging from 10 to 20 mm. COV362, OVSAHO, and TYKNU cells were stained with 10, 15, and 20 mm concentration PKH‐26, respectively. KURAMOCHI and OVCAR8 were stained with 10 mm PKH‐67. Fibroblasts were stained with 10 mm with either of the dyes depending on the cancer cell pair. Cell proliferation was not affected by staining (Fig. S1A). For imaging, a total of 2000 cells per well were seeded onto 384‐well plates (CLS3764; Corning, New York, USA). When grown in D‐CC, different cancer to fibroblast cell ratios were tested (1 : 4, 1 : 2, 1 : 1, 2 : 1, 4 : 1) in combination with BJHTERT or WI38; when culturing with PDFs, only one ratio was used (1 : 1). For I‐CC, cancer cell media was supplemented with 75% fibroblast conditioned media. To maintain consistent cancer cell growth conditions, co‐cultures were grown in respective cancer cell media. Minimal proliferation changes were observed between transformed fibroblasts grown in their recommended media compared to the cancer cell media used in co‐cultures. The proliferation of PDFs 032F and 030F decreased slightly in culture with DMEM/RPMI media; however, metabolic activity as measured by CellTiter‐Glo^®^ (CTG, Promega, Madison, WI, USA) remained unchanged (Fig. S1B). Cell viability was measured on an Ensight plate reader (PerkinElmer, Shelton, CT, USA).

### Conditioned media preparation

To collect CM, cells were plated in T75 culturing flask at a concentration of 1 × 10^6^ cells in 13 mL of respective cancer cell culturing media. Cells were incubated for 72 h to reach 60–90% confluency. Then CM was collected, centrifuged for 5 min at 700 x g, filtered through a 0.2 mm strainer, and aliquots were stored at −80 °C until use.

### Olink targeted 96 oncology II assay and data analysis

Samples were collected from cells in monoculture and D‐CC as described above. Neat (undiluted) samples were analyzed using the Oncology II panel as described by Olink Biosciences (Uppsala, Sweden) [14]. Results from 92 cytokines were provided as Normalized Protein Expression (NPX) values, presented as log_2_ (Tables S2, S3). To assess co‐culture‐specific changes, dual fold change (dualFC) was calculated based on the comparison of both cancer and fibroblast monocultures. To do this, first, a theoretical value was calculated based on monoculture results, which was then compared against co‐culture values from the Olink assay. This allowed us to determine emergent, secretory profiles of co‐cultures while accounting for monoculture effects.

$$\;\text{dualFC}=\frac{\text{mean}\left(\mathrm{NP}X_{\text{cancer monoculture}}+\mathrm{NP}X_{\text{fibroblast monoculture}}\right)/2}{\mathrm{NP}X_{\mathrm{co}‐\text{culture}}}$$

### Immunofluorescent staining and drug treatment

Immunofluorescent (IF) staining was used to determine cancer cell drug sensitivity, to evaluate PDFs marker expression and quantify WISP‐1 effects on cell response to rapalogs. For drug screening, cells were plated in a 1 : 1 cancer to fibroblast cell ratio and exposed to a library of 528 approved and investigational compounds obtained from FIMM HTB [15]. For WISP‐1 effect evaluation, KURAMOCHI, OVCAR8, and OVSAHO cells were resuspended in media containing 0, 10, and 200 pg·mL^−1^ WISP‐1 and seeded to a plate containing drugs of interest. All drugs were added to 384‐well plates (6 007 688, Revvity, Waltham, MA, USA) using an acoustic liquid dispensing system Echo550 (Beckman/Labcyte, Brea, CA, USA). Cells were plated into pre‐spotted drug plates using a Multidrop Combi (Thermo Fisher Scientific) and treated for 72 h in an incubator. Cells were fixed with 4% PFA/PBS (Thermo Fisher Scientific) for 10 min following 10 min permeabilization with 0.3% Triton‐100X/PBS (Thermo Fisher Scientific) and 1 h blocking with PBS containing 3% bovine serum albumin (Sigma‐Aldrich). Plates were incubated overnight at 4 °C with primary antibodies diluted in 1% BSA in 0.1% Tween‐20 (Thermo Fisher Scientific). On the following day, secondary antibodies were added and incubated for 1 h at room temperature. Once done, cells were stained with Hoechst 33342 (Invitrogen) for 15 min at RT. Washes were performed between each step using a Biotek EL405 (AH Diagnostics, Aarhus, Denmark) automated plate washer. Plates were imaged within 1 week after staining. This staining protocol was also used for PDFs marker expression studies, while cells with WISP‐1 were stained for Hoechst 33342 only. Antibodies and concentrations used in all assays are stated in Table S4.

### Imaging

Live‐cell proliferation was measured every 12 h for 72 h using the IncuCyte^®^ S3 system (Sartorius, Germany) (IC) generating a 7 time point image sequence. Images were acquired at 4×, capturing 1 field of view (FOV) in brightfield, red, and green fluorescent channels. The remaining datasets were imaged using the high‐content screening system OperaPhenix (Revvity, USA). Cell morphology images were acquired at 20× using 9 FOV with 3% overlap 72 h after culture. IF stained PDF, cancer monocultures, and co‐cultures were imaged at 10×, capturing 4 FOV with 5% overlap 72 h after drug treatment. Live‐dead *ex vivo* cultures were acquired at 10×, measuring 1 FOV of 20 z‐stack planes at 0 and 72 h after drug treatment.

### Image analysis

Live‐cell proliferation image analysis was done with IC software (Sartorius, Germany) or CellProfiler analyst v4.2.4 [16] (CP) to extract cancer cell covered area based on presence of different dyes. The output modules for IC are called “Total Area (mm2/Image)” and “Confluence%”, while CP output is: “AreaOccupied”.

To analyze cell count and morphological features from monoculture and co‐culture assays, we first stitched all FOV using the Python3 PIL library to create whole well images. These stitched images were then corrected for uneven illumination using CP modules “CorrectIlluminationCalculate” and “CorrectIlluminationApply”. Further, we implemented a deep learning algorithm Stardist2D (2D_versitile_fluo) to segment nuclei, providing the total nuclei count in co‐culture assays. Next, to understand the morphological changes in cancer cells induced by D‐CC and I‐CC conditions, we used CP to extract single‐cell morphological features, including object intensity, size and shape, and granularity, resulting in a total of 318 features per image. The final output contained aggregated average values normalized to DMSO controls representing each well.

Images representing PDF validation experiments were analyzed using Harmony software v5.2 (Revvity). First, nuclei and antibody channels were filtered using Gaussian smoothing to reduce the noise. Further, a nuclei mask was created (method “M”) which was used to find cell cytoplasm (method “B”). Finally, population selection was done to take autofluorescence into account. The final data define the number of nuclei with positive marker expression per well.

*Ex vivo* spheroid cell images were analyzed using Harmony software. Here, the image region representing TMRM and POPO‐1 positive cells was generated, and the volume of spheroids was calculated by subtracting live and dead cell regions. The final output includes features describing the 3D aggregates.

### Data analysis

Data analysis and visualizations were performed using R v4.3.1 and Prism9 v10 (GraphPad Software, Boston, MA, USA). To determine cancer cell mutational profile, gene expression and copy number variation (CNV) data was retrieved from the DepMap portal (22Q2) (https://depmap.org/portal) [17]. To evaluate fold growth of live‐cell proliferation, the 12‐h time point was used as baseline to determine the proliferation change over time. Cell morphology assay batch correction was performed using the “Harmony” v1.2.0 R package. CAFs validation represented marker positive cell percentage based on total nuclei count. Drug sensitivity score was calculated for drug screens using web‐based Breeze software (FIMM, Finland) with the DSS2 method and 4‐parametric logic (4‐PL) curve fitting [18]. Drug screening quality was assessed by calculating Z' (Table S5). Synergy was evaluated by calculating the ZIP synergy score using “synergyfinder” v3.8.2 R package. Statistical analysis was performed in R v4.3.1. The differences between the compared groups were analyzed by two‐sided Mann–Whitney U (Wilcox rank‐sum) test; the results were considered statistically significant when *P* < 0.05. Experimental data were presented as mean ± standard deviation (SD), of two to eight technical replicates depending on the assay.

### *Ex vivo* drug testing

Drug testing was performed as previously described by Åkerlund et al. [12] with several modifications. Briefly, cells after tumor dissociation were viably frozen. For drug testing, cells were thawed and plated into 384‐well plates (Corning) with a media change after 48 h and drug treatment after 5 days post‐plating. Cell treatment was done using Apricot^®^ S3 (SPT LabTech, UK) to lift drugs diluted in media from pre‐spotted plates (Greiner, Austria) on cell plates. Assay plates were imaged at 3 h and 72 h after treatment by assessing live (TMRM) and dead (POPO‐1) cell signals.

### *Ex vivo* spheroid characterization using flow cytometry

After thawing patient samples, a portion of cells was used for flow cytometry to determine cellular composition. First, samples were washed with PBS and dissociated into single cells using TrypLE (Thermo Fisher). Following a 15–30 min incubation, cells were washed with primary cell media (RockiT), followed by a wash in PBS, and finally resuspended in MACS buffer (0.5% BSA, 2 mm EDTA, PBS) at a concentration of ~1 × 10^6^ cells·mL^−1^. The cell suspension was blocked for 10 min with Human TrueStain FcX™ (BioLegend, USA), and 100 ⎧L of the suspension was transferred to a well in a 96‐well plate (Greiner, Austria) and centrifuged. Next, the cells were resuspended in a cocktail of antibodies (Table S4) and incubated for 20 min at 4 °C in the dark. This was followed by a wash with MACS buffer and 7‐AAD stain (BD Biosciences, USA) for 7 min. The cells were then resuspended in 200 ⎧L of MACS buffer. Data acquisition was performed using an Attune NxT flow cytometer with CytKick autosampler (ThermoFisher, USA) and analyzed using FlowJo v10.6.0 software (BD Biosciences). Fluorescence minus one (FMO) controls were used to correct for spectral overlap, while matching isotype controls were included to determine background signal. Forward and side scatter were used to gate single cells. The live‐cell population was determined based on the 7‐AAD marker (BD Biosciences). For further gating, all live cells were assessed for CD45 and EpCam expression. CD45 + EpCam‐ cells were gated for all remaining markers to determine the leukocyte population. CD45‐EpCam+ cells were gated for all remaining markers to identify EpCam+CD24 ± populations, while EMT‐like cells were expressing EpCam+ and PDPN or CD90 markers. Finally, CD45‐EpCam‐ cells were gated to determine endothelial cells (CD31+), mesothelial (PDPN+) and stromal (CD90+) cell populations.

## Results

### Cancer cell proliferation and morphology are altered in the presence of fibroblasts

To investigate the interplay between cancer cells and fibroblasts, we selected a panel of cell lines representative of HGSOC based on mutational, gene expression, and CNV profiles from publicly available data [17, 19] encompassing relevant molecular features of HGSOC. We ensured the use of cell lines that carry a mutation in *TP53* (Fig. S1C). In addition, the cancer cells used in this study have different homologous recombination deficiency (HRD) statuses (Fig. S1D,E gene list assessed in Table S6). Among the chosen cell lines, KURAMOCHI and COV362 showed the most impaired HRD, and TYKNU the least. The cell line panel presents a heterogeneous gene expression profile (Fig. S1F) and CNVs in genes associated with HGSOC (Fig. S1G). To comprehensively explore the impact of fibroblast origin, we used both commercially available non‐cancerous fibroblasts (NF) and PDFs isolated from primary patient OC tissues.

The purity of the established PDFs was validated through marker expression profiling and confirmed by the absence of OC‐associated mutations. All fibroblasts expressed alpha‐smooth muscle actin (α‐SMA), vimentin (VIM), and fibroblast‐specific protein‐1 (FSP‐1), albeit at varying levels (Fig. S1H,I). While high levels of FSP‐1 were observed in all fibroblasts, only WI38 and 032F cells showed high expression of both α‐SMA and VIM (≥45%). The 030F cells were positive for VIM, but not α‐SMA (35%). Interestingly, BJHTERT and 042F cells exhibited high α‐SMA, but low VIM (15% and 44% respectively). Notably, only the 042F cells expressed high levels of epithelial cell marker CK8/18. Given that CAFs can derive from different cell types of origin [20], the unique 042F marker expression profile suggests it may represent a distinct fibroblast subtype originating from epithelial cells and undergoing epithelial‐mesenchymal transition (EMT). In addition, our findings are supported by another study reporting that OC fibroblasts may exhibit epithelial cell marker expression, including cytokeratin 8, 18, and 19 [21]. Despite the varying marker expression, somatic mutations found in the corresponding cancer tissue or ascites fluid were absent in the PDF populations, confirming their non‐cancerous origin (Fig. S1J). Additionally, PDFs used in this study were derived from patients diagnosed with different OC subtypes (Table S1), potentially accounting for the observed heterogeneity in marker expression.

To understand the effect of contact between cancer and fibroblast cells, we established a flexible co‐culture assay based on live‐cell imaging. Using this method, we investigated both direct (Fig. S1K) and indirect (Fig. S1L) co‐culture effects, monitoring cancer cell proliferation dynamics over 72 h and revealing different dependency patterns. Specifically, COV362 cells exhibited increased growth in D‐CC as compared to monoculture (FC = 1.32, *P* = 0.0029 at a 1 : 1 ratio) after 72 h, whereas OVCAR8 proliferation decreased in D‐CC (FC = 0.76, *P* = 0.0002 at a 1 : 1 ratio; Fig. 1B). Only TYKNU cells showed a statistically significant change in I‐CC compared to monoculture (*P* = 0.0018), although the changes were not substantial (FC = 0.9). Conversely, KURAMOCHI and OVSAHO cells did not show any significant proliferation changes in co‐culture (FC = 0.93 and 1.1, respectively at a 1 : 1 ratio). In addition, for most tested cell lines, proliferation effects were not dependent on the cancer‐fibroblast cell ratio. However, a gradual increase in proliferation was observed in OVSAHO and WI38 D‐CC. Here, OVSAHO cell proliferation doubled at a higher fibroblast ratio (FC = 4 at 1 : 4 D‐CC of cancer:fibroblast respectively, and FC = 1.9 in OVSAHO monoculture). However, this trend was not observed in OVSAHO co‐culture with other fibroblasts (Fig. S1K). Additionally, the observed effects appeared to be independent of fibroblast origin, as the changes varied predominantly based on the cancer cells. Altogether, direct contact with fibroblasts had more pronounced effects on cancer cell proliferation compared to indirect contact (Fig. 1C).

In addition to changes in proliferative capacity, we observed profound alterations in cancer cell morphology in the co‐culture environment. There are distinct changes in cell cytoplasm and nuclei size, as well as cell colony compactness upon D‐CC (Fig. 1D). To quantify this, we performed dimensionality reduction analysis of 318 cell morphological features, revealing unique patterns for each cancer cell line. In most cases, monocultured and I‐CC cells tend to cluster together (Fig. 1E, Fig. S1M). While TYKNU, COV362, and KURAMOCHI monocultures cluster with I‐CC cells separately from D‐CCs, OVCAR8 and OVSAHO exhibited cluster overlap between culture conditions. In addition, we quantified deviations in feature space between conditions, where COV362 and OVSAHO cell morphological features in D‐CC were significantly different compared to both monocultures and I‐CC, while OVCAR8 cell morphology significantly differed between D‐CC and I‐CC, but not monoculture (Fig. 1F). Interestingly, TYKNU cell morphology changes were small, and while we see D‐CC cluster separated from monoculture and I‐CC, the distances between clusters are small. We also observed a weak negative correlation between morphological profile and proliferation changes (Fig. S1N). Despite the variable CAF marker expression between fibroblasts, we did not find any significant differences in their morphology; 042F morphology was similar to the remaining PDFs, as well as the NFs (Fig. S1O). Altogether, cell proliferation and morphology alterations showed a weak correlation, with cells exhibiting the most altered proliferation levels (COV362 and OVCAR8) morphology in D‐CC. Thus, our findings underscore the impact of direct interaction between fibroblast and cancer cells on both proliferation and morphology levels. In our study, these alterations were independent of fibroblast origin, but rather shaped by the cancer cells.

### Cytokine profiling reveals unique co‐culture effects

**Fig. 2:**
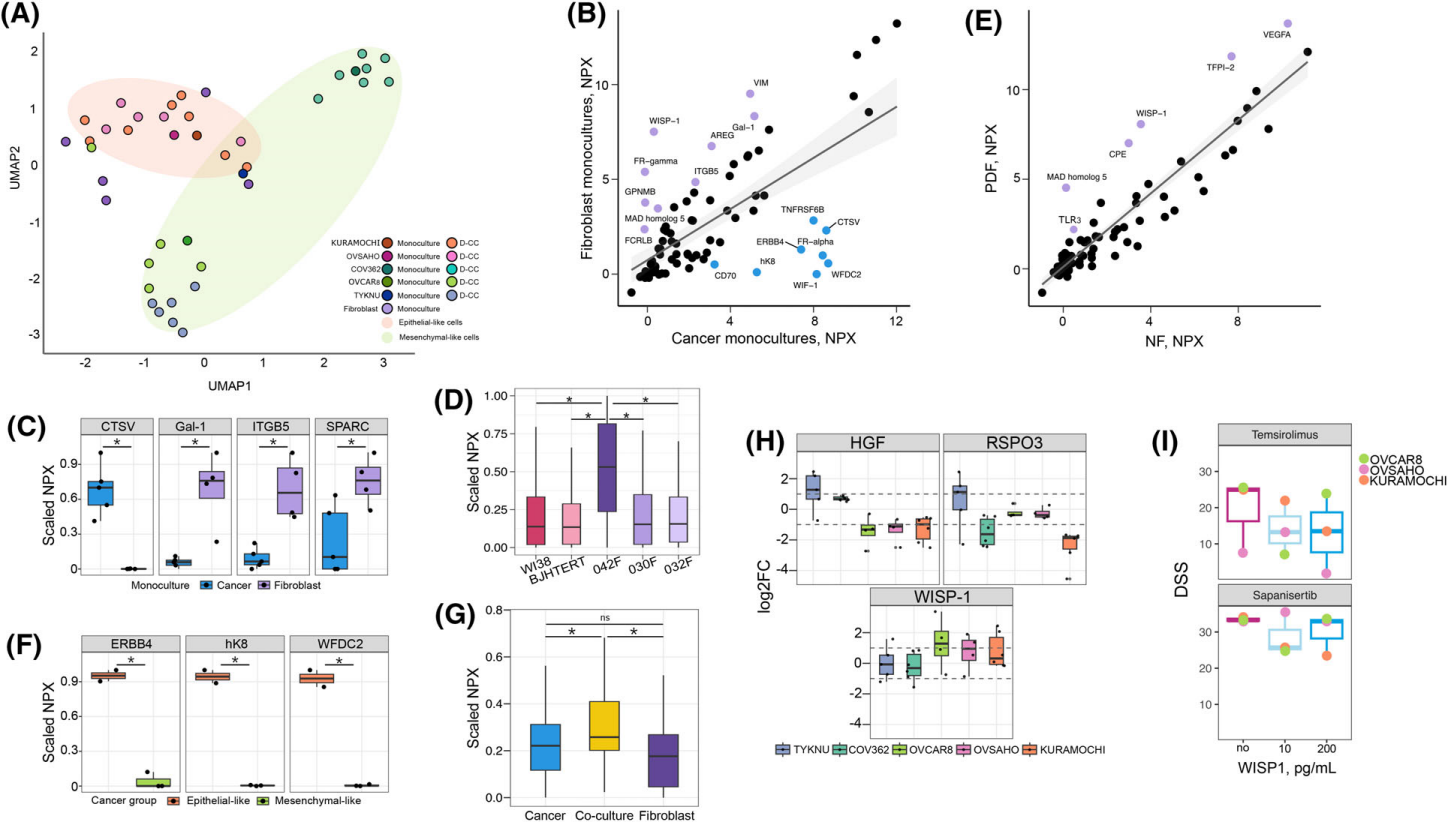
Cytokine profiling reveals differences between monocultures and co‐cultures. (A) UMAP plot of the secretory profile of monocultured and co‐cultured cells. Points are colored by culture condition according to legend; the light‐colored ellipses circles mark epithelial (red)/mesenchymal (green)‐like cancer cells in the plot. (B) Scatterplot highlighting differences in secretory profile between cancer and fibroblast cell monocultures for the 92 cytokines profiled. Each dot is the average normalized protein expression (NPX) for the cell population. (C) Boxplot showing cytokines with statistically significant differences between cancer and fibroblast monocultures, shown as scaled NPX (0 to 1), *P < 0.05 from Mann–Whitney U test. (D) Boxplot representing the differences of overall cytokine secretion between fibroblast models used in this study, shown as scaled NPX (0 to 1), *P < 0.05 from Mann–Whitney U test. (E) Scatterplot highlighting the secretory profile differences between non‐cancerous (NF) and patient‐derived (PDF) fibroblast monocultures, for the 92 cytokines profiled. Each dot is the average NPX for the cell population. (F) Boxplot showing differences of cytokine secretion between epithelial/mesenchymal‐like cancer cell monocultures, shown as scaled NPX (0 to 1), *P < 0.05 from Mann–Whitney U test. (G) Comparison of secretory profile between the two monocultures and co‐culture conditions, shown as scaled NPX (0 to 1), *P < 0.05 from Mann–Whitney U test. (H) Boxplot representing the most differentially secreted cytokines between co‐cultures and monocultures from both cell types, expressed as dualFC, *P < 0.05 from Mann–Whitney U test. (I) Boxplot representing drug sensitivity without and with WISP‐1 supplemented media.

Since we observed stronger effects of D‐CC compared to I‐CC, we performed cytokine profiling of 92 molecules using the Olink Biosciences platform on D‐CCs and monocultures to further dissect the molecular crosstalk between these two populations. This analysis revealed significant cytokine alterations with distinct signatures dependent on the cancer cells in culture (Fig. 2A; Fig. S2A). Hierarchical clustering showed that KURAMOCHI and OVSAHO cells group together based on secretory profile, while TYKNU cells clustered with fibroblast monocultures. Notably, based on the analysis of gene expression data from DepMap portal [17], we observed that KURAMOCHI and OVSAHO have higher levels of epithelial‐like cell marker expression compared to the other cancer cell lines (Fig. S2B; Table S6). In addition, projection of metastatic and invasiveness gene sets shows that OVCAR8, TYKNU, and COV362 have higher EMT properties than KURAMOCHI and OVSAHO (Fig. S2C). These differences suggest that EMT status and underlying gene expression signatures may influence the observed secretory profiles. We also identified differences in cytokine secretion between cancer and fibroblast cells (Fig. 2B). Among those, Cathepsin V (CTSV) (log_2_FC = 7.2, *P* = 0.002), a protease involved in ECM degradation [22], was secreted exclusively by cancer cells. In contrast, fibroblast monocultures demonstrated significantly higher levels of galectin‐1 (Gal‐1) (log_2_FC = 3.0, *P* = 0.029), integrin beta‐5 (ITGB5) (log_2_FC = 2.1, *P* = 0.017) and secreted protein acidic and rich in cysteine (SPARC) (log_2_FC = 1.6, *P* = 0.019), all of which play a role in cell–cell and cell‐ECM interactions [23, 24, 25] (Fig. 2C; Fig. S2D). Among the fibroblasts, 042F showed significantly higher levels of total cytokine secretion (Fig. 2D). PDF cytokine secretion comparison indeed showed that 032F and 030F have more similar profiles (Fig. S2E), as compared to 042F (Fig. S2F). Considering differences in 042F cytokine secretory profile and its distinct CAFs marker expression profile, 042F was excluded from subsequent comparisons in Fig. 2E,F. The remaining PDFs (032F and 030F) showed increased secretion of cytokines associated with metastasis and disease dissemination (ANAX1, CPE, GPNMB, VEGFA, VIM, WISP‐1) [26, 27, 28], as compared to NF. In addition, PDFs exhibited higher secretion of cytokines associated with pleotropic tumorigenic effects (SMAD5, TLR3, TFPI‐2) [28, 29] (Fig. 2E). We also observed differences in the secretory profile of epithelial‐like (KURAMOCHI and OVSAHO) and mesenchymal‐like (COV362, OVCAR8, TYKNU) cancer cells. Epithelial‐like cells showed significantly higher secretion of OC‐associated proteins, such as erb‐b4 receptor tyrosine kinase (ERBB4) (log_2_FC = 4.5, *P* = 0.002), kallikrein‐8 (hK8) (log_2_FC = 6.2, *P* = 0.038) and epididymis protein‐4 (WFDC2) (log_2_FC = 7.1, *P* = 0.049) (Fig. 2F; Fig. S2G). ERBB4 and hK8 are linked to better OC outcomes [30, 31], while WFDC2 may contribute to alterations in the TME leading to disease progression [32]. A global comparison showed significantly higher levels of secretory cytokines in co‐cultures compared to both monocultures (cancer monoculture *P* = 0.002, fibroblast monoculture *P* < 0.0001) (Fig. 2G). To investigate co‐culture‐specific changes, a dualFC calculation was performed. This analysis revealed that hepatocyte growth factor (HGF), WNT1‐inducible signaling pathway protein 1 (WISP‐1), and R‐spondin (RSPO3) showed co‐culture‐specific effects, although these were not statistically significant (Fig. 2H; Fig. S2H). KURAMOCHI, OVSAHO, and OVCAR8 cell co‐cultures displayed lower HGF secretion and increased WISP‐1 levels compared to both monocultures. Both of these proteins are related to growth in multiple cancer types including OC [33, 34]. Further validation confirmed increased WISP‐1 levels in co‐cultures of KURAMOCHI, OVSAHO, and OVCAR8 as detected in the Olink assay (average dualFC = 1.45) (Fig. S2I). To further understand the functional impact of WISP‐1, KURAMOCHI, OVSAHO, and OVCAR8 cells were exposed to high and low WISP‐1 concentrations (10 and 200 pg·mL^−1^). However, WISP‐1 alone did not significantly alter cell proliferation (Fig. S2J). Nevertheless, WISP‐1 altered cell response to inhibitors targeting mTORC1/2, one of the pathways activated by WISP‐1 [35]. KURAMOCHI and OVCAR8 showed high sensitivity to Temsirolimus (mTORC1 inhibitor) and Sapanisertib (mTORC1/2 inhibitor), while OVSAHO showed lower sensitivity to Temsirolimus than Sapanisertib (Table S7). In the presence of WISP‐1 (10 or 200 pg·mL^−1^), the cancer cells showed increased resistance to these drugs. Temsirolimus showed a decreased cytotoxicity in all cell lines in the presence of WISP‐1; however, the impact of different WISP‐1 concentrations varied across the three cancer cell lines. KURAMOCHI was more resistant to Temsirolimus with increasing WISP‐1 concentrations (DSS 25 with 10 and 10 with 200 pg·mL^−1^ WISP‐1) OVSAHO cells were most resistant to Temsirolimus in the presence of 200 pg·mL^−1^ WISP‐1 (DSS 7.5 without WISP‐1 and 0.3 with 200 pg·mL^−1^ WISP‐1), while OVCAR8 cells were more resistant to Temsirolimus at lower WISP‐1 concentrations (DSS 24.2 without WISP‐1 and 6.6 with 10 pg·mL WISP‐1). While the response to Sapanisertib showed similar trends, the effect of the drug remained high in all conditions (DSS > 20) (Fig. 2I). These observations suggest that WISP‐1 may play a role in increased drug resistance in cancer cells and alter the mTOR pathway, a central regulator of cell growth, survival, and metabolism. In summary, using a 92 cytokine panel, we identified several altered cytokines in co‐culture conditions and the secretory profiles were unique to each co‐culture.

### A co‐culture screen highlights the impact of fibroblasts on drug response

**Fig. 3:**
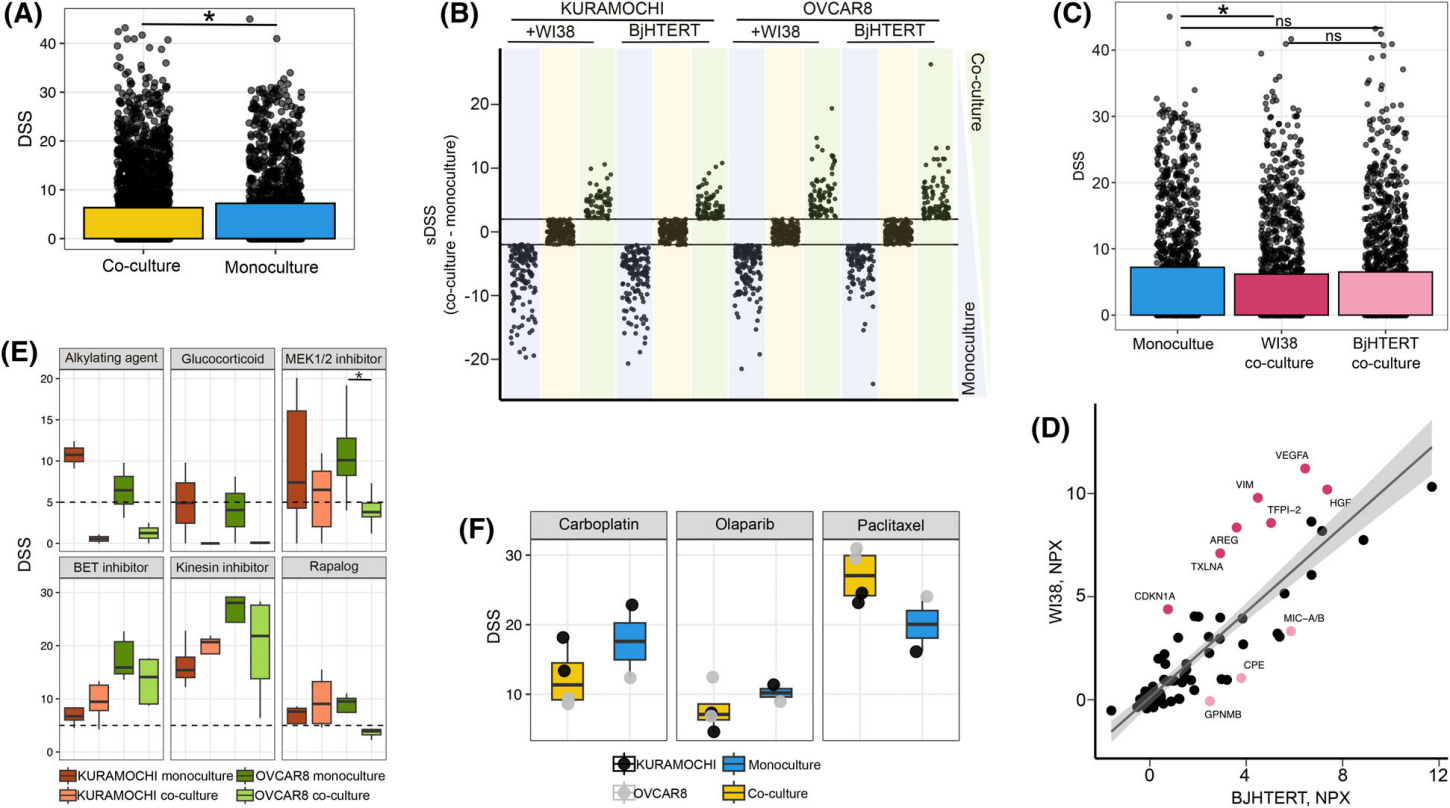
Co‐culture screening reveals cancer cell resistance to drugs in presence of fibroblasts. (A) Drug response comparison between cancer cell monocultures and co‐cultures, expressed as drug sensitivity score (DSS), *P < 0.05 from Mann‐Whitney U test. (B) Distribution of drugs sensitizing differentially co‐cultures and monocultures, lines indicate selective DSS (sDSS) ± 2. (C) Comparison of drug response between both cancer monocultures and co‐cultures with BJHTERT and WI38 fibroblasts, *P < 0.05 from Mann–Whitney U test. (D) Comparison of BJHTERT and WI38 monoculture cytokine secretion levels, highlighting cytokines that are differentially secreted between the cells. (E) Drug subclasses exhibiting the most altered drug response depending on TME conditions, *P < 0.05 from Mann–Whitney U test. (F) Boxplot representing KURAMOCHI and OVCAR8 monoculture and co‐culture drug response to standard‐of‐care treatment.

To further determine the impact of co‐culture on cancer cell drug sensitivity, we performed a high‐content imaging‐based screen to compare drug responses in cancer cell monocultures and D‐CC. KURAMOCHI, a less aggressive disease model, and OVCAR8, a more aggressive one, were selected for drug testing based on their molecular characteristics and compatibility with the assay (Fig. S3A). Using IF imaging, we were able to distinguish the two cell populations in co‐culture and evaluate their response to 528 drugs (Table S8). A comparison between monocultures and co‐cultures revealed significantly higher (*P* = 0.018) drug sensitivity in cancer cell monocultures (Fig. 3A), which suggests that fibroblasts, as one of the TME components, induce chemoresistance [36]. We also observed a larger drug response distribution in cancer cells in co‐cultures, suggesting a heterogeneous response depending on context. We found that 65% (*n* = 335) of the drugs had increased sensitivity in at least one of the culture conditions (drug response score (DSS) < 4). Among the tested drugs, 28% showed greater efficacy in cancer cell monocultures, 17% in co‐cultures, and 55% did not show a shift in drug efficacy despite changes of TME (selective DSS (sDSS) > 2, Fig. 3B). Interestingly, cancer cells in co‐culture with WI38 fibroblasts exhibited significantly higher drug resistance (*P* = 0.002), while the presence of BJHTERT fibroblasts had a less pronounced effect (*P* = 0.27). While both co‐cultures showed reduced cancer cell drug sensitivity, KURAMOCHI cells had a greater decrease in sensitivity compared to OVCAR8 (sDSS = 1.25 and 0.49 respectively) (Fig. 3C; Fig. S3B). Notably, we found that WI38 secreted higher levels of several cytokines as compared to BJHTERT (Fig. 3D; Fig. S3C), which may contribute to the observed differences in drug response. Cancer cells in co‐culture with BJHTERT were more sensitive to MEK, PI3K, and mTOR inhibitors (Fig. S3D). These findings could be linked to the secretion of GPNMB by BJHTERT cells, a protein known to upregulate the PI3K pathway and potentially alter the response to PI3K inhibitors [37]. In contrast, WI38 secreted cytokines such as HGF and AREG, which are known to activate MEK, PI3K, and mTOR pathways. Indeed, cancer cells in co‐culture with WI38 demonstrated increased resistance to inhibitors targeting these pathways. This suggests the presence of alternative mechanisms in cancer cells that confer protection against these drugs. Resistance to MEK inhibitors has been associated with HGF in melanoma [38] while resistance to PI3K inhibitors has been associated with AREG, among other factors [39].

The drug library used in this study consists of eight drug classes representing 49 drug subclasses based on mechanism of action (MoA) (Table S9). Some MoA classes are represented more heavily than others, with the smallest subclass containing two drugs and the largest over 30. Nevertheless, we have observed several trends (Fig. S3E,F). Monocultures were the most sensitive to conventional chemotherapy. Among the different MoAs, alkylating agents, glucocorticoids, and MEK1/2 inhibitors exhibited killing effects in both KURAMOCHI and OVCAR8 monocultures. Interestingly, KURAMOCHI cells in co‐culture were more sensitive to rapalogs, BET, and kinesin inhibitors, while OVCAR8 cells in co‐culture exhibited higher resistance to these subclasses of drugs. Several other drug subclasses demonstrated differences between monoculture and co‐culture conditions (Fig. 3E; Fig. S3G); however, these differences were not significant. Notably, drugs used in standard OC management were also affected by co‐culture conditions (Fig. 3F; Fig. S3H). Cancer cells in the presence of fibroblasts showed higher resistance to Carboplatin treatment (overall sDSS = 5.2). Olaparib, used for HRD OC treatment, decreased response in KURAMOCHI cells from DSS = 11.4 in monoculture to 5.95 in co‐culture (sDSS = 5.45). Interestingly, even though OVCAR8 monoculture response to Olaparib was weaker, cancer cells in co‐culture were not as affected as KURAMOCHI (sDSS = 0.7). Finally, Paclitaxel response was increased upon co‐cultures in both cancer cell lines (average sDSS = 7). This drug targets fast‐proliferating cells; however, our proliferation assay showed the contrary – proliferation (measured by area occupied) decreases. These results suggest that the presence of fibroblasts increases cancer cell susceptibility to this drug through alternative mechanisms. Altogether, we have observed a decreased response of cancer cells to drug treatment in co‐culture, including drugs used in clinical settings.

### Vorinostat and Birinapant as potential combination treatment for OC

**Fig. 4:**
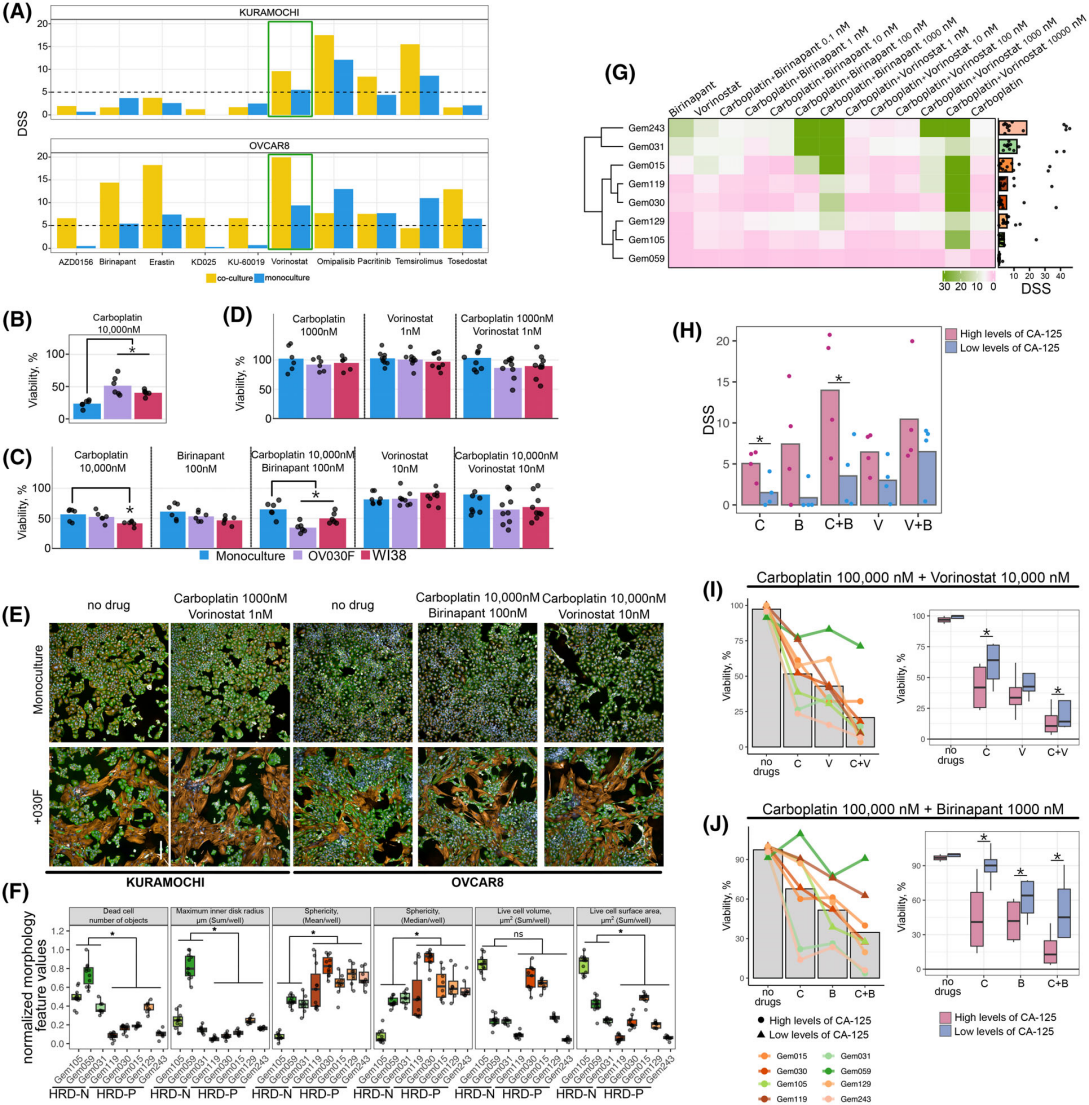
Vorinostat and Birinapant in combination with Carboplatin show synergistic effects in cell co‐cultures and *ex vivo* spheroids. (A) A set of drugs that showed selective drug response to cancer cells of KURAMOCHI or OVCAR8 cells in co‐culture. Green rectangle marks Vorinostat, a drug that inhibited both tested cancer cell growth in co‐cultures. Subsequent validation experiment results, representing KURAMOCHI and OVCAR8 cancer cell response in monocultures and co‐cultures. Bars show mean viability after 72 h treatment, each dot represents biological and technical replicates (*n* = 9), *P < 0.05 from Mann–Whitney U test: (B) response to Carboplatin at 10000 nm concentration, (C) OVCAR8 response to Carboplatin, Vorinostat, Birinapant single drug treatment and their combination, (D) KURAMOCHI response to Carboplatin, Vorinostat, single drug treatment and their combination. (E) immunofluorescent images representing cell co‐cultures of cancer cell monocultures and co‐cultures with 030F, with and without treatment. Green in the images represents cancer cells, stained with CK8/18, and orange – fibroblasts stained with vimentin, blue – nuclei stained with Hoechst, scale bar 200 μm. (F) Morphological features of *ex vivo* spheroids generated from patient material, box plots indicate differences between each sample, dots represent technical replicates (*n* = 12), *P < 0.05 from Mann–Whitney U test. (G) Heatmap overview of patient *ex vivo* response to Vorinostat, Carboplatin, and Birinapant as single agents and combination treatments. Unsupervised sample hierarchical clustering performed using the Euclidean distance metric. The bar plot on the side represents the average of overall drug response. (H) Sample drug response comparison between the CA‐125 high and low level groups, dots represent each patient, *P < 0.05 from Mann‐Whitney U test. Here C corresponds to Carboplatin, V – Vorinostat, B – Birinapant. Sample drug response when treated with single drug and combinations. Bar on the left side represents each sample viability change when treated; bars indicate average values. While boxplots on the right represent the differences between CA‐125 high and low groups viability under treatment (*n* = 3), *P < 0.05 from Mann–Whitney U test: I – represents results after 100 000 nm Carboplatin and 10 000 nm Vorinostat drug treatment; J – represents results after 100 000 nm Carboplatin and 1000 nm Birinapant drug treatment. Here C corresponds to Carboplatin, V – Vorinostat, B – Birinapant.

Although the drug screening did not identify specific drug classes that consistently affect cancer cells under systematic change of TME, our data revealed several drugs that were more effective at targeting cancer cells in co‐culture conditions. Specifically, we selected drugs or small molecules that exhibit low response in monocultures (DSS < 10), but had a greater impact in co‐cultures (DSS >5 with sDSS≥4). This approach led to the identification of 10 drugs that displayed robust drug response curves and differential sensitivity depending on co‐culture conditions (Fig. 4A). Interestingly, most of these drugs had opposite effects on KURAMOCHI and OVCAR8 cell lines. However, one drug, HDAC inhibitor Vorinostat, significantly sensitized both cancer cell lines in co‐culture (sDSS = 4.1 and 10.55, respectively) in the screen (Fig. S4B). These effects were also visible in fluorescent images (Fig. S4A). Additionally, Birinapant showed strong effect in OVCAR8 co‐cultures (sDSS = 9.0) (Fig. S4C). Further analysis using ssGSEA pathway enrichment from the DepMap portal [17] revealed that both KURAMOCHI and OVCAR8 cell lines showed upregulation in the HDAC targets (enrichment scores of 0.15 and 1.7, respectively), suggesting that both cell lines are inherently more responsive to HDAC inhibitors. In contrast, the SMAC‐XIAP‐regulated apoptotic response pathway was enriched only in the OVCAR8 cell line (enrichment score of 0.11), which is in line with our drug response data (Fig. S4D). Of genes in these pathways, HDAC2 and TNFRSF1, respectively, correlated the best with the observed response patterns, and future studies investigating these as potential biomarkers would be of interest (Fig. S4E).

Given that Vorinostat and Birinapant were more effective in co‐cultures, we sought to explore their combination with Carboplatin as a strategy to improve the standard therapy regimen. To evaluate this, we tested the effects on cancer cells in co‐cultures with WI38 and 030F, fibroblasts derived from HGSOC (Table S10, Fig. S4F). Carboplatin treatment showed increased sensitivity in KURAMOCHI cells, with significant differences at 10000 nm Carboplatin concentration, resulting in 24% viability in monoculture and 47% in co‐cultures (*P* = 0.0001) (Fig. 4B). However, OVCAR8 cells did not show such significant differences, with an average viability of 50% in all conditions (Fig. S4G). Surprisingly, in these validation studies, both OVCAR8 and KURAMOCHI did not demonstrate significant differences in drug response between culture conditions when treated with Vorinostat or Birinapant (Fig. S4H,I), unlike previously shown. We observed that the complexity of cancer and fibroblast culture was hard to control between biological replicates. Despite rigorous cell counting, we found that cancer and fibroblast cell ratios between separate biological replicates varied; one replicate maintained an equal ratio of cancer and fibroblast cells after 72 h in culture, while the two other replicates had an increased ratio of cancer cells in co‐culture (Fig. S4J,K). Importantly, both WI38 and 030F fibroblasts’ marker expression was consistent throughout the experiments (Fig. S4L). Nevertheless, our data revealed increased drug sensitivity to the drug combinations in co‐culture conditions. Treatment with 10 000 nm Carboplatin and 100 nm Birinapant combination showed significant decrease in OVCAR8 cell viability in co‐culture (*P* = 0.004) leading to an average viability of 42%. Vorinostat treatment was less effective; however, the combination of 10 000 nm Carboplatin and 10 nm Vorinostat resulted in a 23% difference in cell viability between culture conditions in OVCAR8 cells (Fig. 4C) Although KURAMOCHI did not show such strong sensitivity to this drug combination, the combination of 1000 nm Carboplatin and 1 nm Vorinostat decreased cancer cell viability by 17% compared to monocultures (Fig. 4D). Overall, co‐culture systems highlight the challenges of such experimental design but also show that cancer cells are indeed affected by the presence of fibroblasts and are important in shaping cancer cell biology.

To further explore the potential of Carboplatin combinations with Vorinostat or Birinapant, we used patient material to better mimic *in vivo* conditions. We used 3D *ex vivo* cultures from dissociated tumor tissue of representative HGSOC patients with similar clinical stage at diagnosis from our biobank (Table S11) and applied the DET3CT platform [12] to determine drug response. We selected a total of eight patients presenting with advanced stage HGSOC and a range of CA‐125 levels (160–7370 U·mL^−1^). In addition, these samples also represent both positive (HRD‐P: Gem119, Gem030, Gem015, Gem129, Gem243) and negative (HRD‐N: Gem105, Gem059, Gem031) HRD status. Interestingly, the HRD status appears to influence the morphology of the spheroids (Fig. S4M). On average, HRD‐P samples formed smaller but more rounded spheroids as compared to HRD‐N samples (Fig. 4F). Our image analysis identified differences between HRD‐N and HRD‐P patient spheroid morphology, suggesting an intrinsic link between HRD status and spheroid architecture.

*Ex vivo* drug treatment showed that most of the patients did not respond well to monotherapy with Carboplatin, Vorinostat, or Birinapant (average DSS = 3.3, 4.7, 4.2 respectively) (Table S12, Fig. G). While Gem243 and Gem031 were the most sensitive to single drug treatment (average DSS 9.7 and 7.2 respectively), the remaining samples did not show a significant response (DSS <5). In addition, the cellular composition varied among patient samples (Fig. S4N). While Gem119 exhibited a high proportion of CD45+ cells (95% of live cells), the remaining samples contained a higher percentage of cancer cells (at least 45% of all live cells). In addition, all samples, except for Gem015, contained a stromal cell compartment, although it did not exceed 13%. Interestingly, our data revealed a correlation between the presence of an EMT‐like cancer cell population and elevated levels of CA‐125, while no strong correlation was observed between CA‐125 levels and other EpCam+ cell populations (Fig. S4O). Overall, the high CA‐125 sample group showed higher sensitivity to the tested drugs, particularly for the Birinapant and Carboplatin combination (*P* = 0.013) (Fig. 4H). To evaluate drug combination response, the ZIP synergy score was calculated (Table S13). Overall high synergy (ZIP>10) was observed in Gem105 for both Vorinostat and Birinapant combination with Carboplatin (ZIP 14.8 and 12.4 respectively). Gem031 and Gem015 showed antagonistic (ZIP > −10) drug combination effects for the Vorinostat combination. Notably, these samples had some of the highest single agent responses to Vorinostat. For all the remaining samples, an additive effect (−10 < ZIP<10) was observed. Drug treatment demonstrated strong effects at higher concentrations in both drug combinations (Fig. S4O,P, Table S14). At 100 000 nm Carboplatin and 10 000 nm Vorinostat, cell viability dropped below 30% in most samples, exhibiting greater killing compared to both single drug effects, where viability was 48 and 37% for Carboplatin and Vorinostat respectively (Fig. 4I). Although the combination of 100 000 nm Carboplatin and 1000 nm Birinapant exhibited less killing (35% on average), enhanced killing was observed across all samples at these drug concentrations, with the exception of Gem059 (Fig. 4J). Treatment with 100 000 nm Carboplatin and 1000 nm Birinapant led to a cell viability decrease to 26.7%, compared to 48 and 62% viability for the single drugs, respectively. In addition, both drug combination treatments showed greater efficacy in samples with high CA‐125 levels, particularly with the Birinapant and Carboplatin combination, where the difference between the groups was threefold.

Overall drug combination treatment showed increased cell death, especially for the sample group with high CA‐125 levels; however, the response is dependent on the dose. Our results warrant further study in a larger patient cohort to refine understanding of the patient characteristics that predict a favorable response to such treatment.

## Discussion

In this study, our aim was to understand the effects of fibroblasts on OC cell behavior and drug response. Increasing attention is being directed towards the impact of TME on cancer progression and response to treatment [40]. CAFs, as a critical component of TME, have been shown to influence OC progression and chemoresistance. While efforts have been made to target cancer dependencies on supportive CAFs signaling, unfortunately, many challenges remain [10]. Thus, it is crucial to understand how cancer cells change in the presence of CAFs and to identify novel vulnerabilities that emerge upon this interaction.

Our data indicate that cancer cells respond heterogeneously to CAFs, leading to changes in cell proliferation and morphology. We observed both an increase and decrease in cancer cell proliferation, predominantly under D‐CC conditions, suggesting that cell–cell contact has a greater impact than fibroblast secreted factors alone. Across literature studies, there are conflicting results regarding the impact of CAFs on cancer cell proliferation and growth. For instance, contact‐independent effects were shown to lead to proliferation changes in colon cancer cells [41]; however, in melanoma cancer cells, CM increased invasion but not proliferation [42]. The observed variations likely depend on the experimental setup, as factors secreted by fibroblasts alone differ from those when fibroblasts are exposed to cancer cells [43]. This in part can explain the weaker effects of fibroblast CM in our study. Cancer cell morphological plasticity and heterogeneity are linked to disease prognosis and aggressiveness [44]. Hence, TME plays a role in altering cancer cell morphology that cannot be mimicked in monoculture settings. For instance, fibroblasts directly influence melanoma cancer cells' phenotypic flexibility and stemness, which leads to more aggressive disease [45]. In this study, we focused on changes in cell morphology, which have been shown to be a powerful way to study cancer, exposing cancer cell states [46]. Our data revealed a clear shift in cancer cell morphology under D‐CC conditions. In contrast, morphological features remained similar to monoculture upon I‐CC. Importantly, the morphological shift in co‐cultures was cancer cell line dependent, showing the complexity and unique trajectory of the disease to individual genetic and phenotypic traits. The supportive role of CAFs in cancer progression also enables cancer cell survival upon treatment. By altering physical ECM features and secreting cytokines, CAFs communicate with cancer cells [47]. For example, secretion of VEGFA promotes angiogenesis and tumor growth, IL6 activates the STAT/JAK1 pathway enhancing cancer cell plasticity, and TGF‐β induces EMT, cell invasion capacity and resistance to treatment [9]. Our data showed the presence of these cytokines in fibroblast monocultures, with significantly higher levels of VEGFA and TGF‐β in PDFs, cells educated *in situ*, indicating their activated, and cancer‐promoting capabilities. In addition, PDFs secreted higher amounts of ITGB5 and SPARC, both of which are known to facilitate EMT and ECM remodeling [24, 48], as well as Gal‐2, which contributes to tumor immunosuppression [49, 50]. Importantly, variability in cytokine secretion profiles among fibroblasts may be influenced by their origin. Previous studies have reported differences between NF and PDFs at transcriptomic [51] and cytokine secretion [52] levels, highlighting the impact of fibroblast origin in their role in TME. Fibroblast origin has also been reported to influence drug response in co‐culture [53]. In this study, we include both NF and PDFs, enabling further comparison between these models. The PDFs, in turn, were generated from patients with different ovarian cancer diagnosis and stage, which can additionally account for the variability in marker expression, morphological characteristics and cytokine secretion profiles. Notably, only one cytokine; CTSV was highly secreted by cancer cells only. This protein has been shown to play a role in ECM degradation and cell invasion with high levels associated with poor outcome in various cancer types [54, 55, 56]. We observed a number of differentially secreted cytokines between cancer and fibroblast cell monocultures; however, the secretory profiles in co‐cultures were more complex, revealing unique patterns for each cancer cell line–CAF combination. For instance, we found elevated levels of tumor‐promoting HGF under D‐CC conditions. HGF/MET signaling is linked to increased proliferation and treatment resistance, and serum HGF levels were correlated with overall survival in OC patients in previous studies [57]. Interestingly, the increase of HGF levels was observed only in more aggressive mesenchymal‐like cell (TYKNU and COV362) co‐cultures. Additionally, RSPO‐3 levels increased in TYKNU and decreased in epithelial‐like KURAMOCHI co‐cultures. Heightened levels of this protein expression are also associated with cancer progression [58]. Finally, three cancer cell co‐cultures showed increased WISP‐1 levels, a protein activating multiple cellular pathways leading to tumorigenesis and disease progression [33, 59]. While presence of extracellular WISP‐1 in culture media did not directly affect cell proliferation, it increased resistance to mTORC1/2 inhibitors across multiple cell lines. Intracellular WISP‐1 has been linked to tumor progression and poor disease prognosis in several cancers [35, 60, 61]; however, the role of extracellular WISP‐1 remains poorly understood. Our findings indicate that WISP‐1 alone may contribute to increased resistance to certain drug treatments, though further mechanistic studies are needed to fully elucidate its impact. Overall, while all identified cytokines are related to progressive disease, the unique cytokine profiles for each cancer cell line suggest a complex interplay between cytokines and determine disease outcomes. This suggests that studies using autologous samples from individual patients may be needed to shed light on the distinct mechanisms active in individual patient's disease progression.

The presence of fibroblasts and their secreted factors contribute to cancer cell drug resistance. To systematically identify drugs that are more effective in the co‐culture setting, we performed a large‐scale drug screen of 528 drugs at 5 dose concentrations. Our data showed the increased resistance to nearly a one‐third of the drugs in co‐culture conditions, consistent with observations in colorectal, pancreatic, breast, and melanoma cancer cells [62]. Alkylating agents were the most affected by environment, showing increased resistance in co‐cultures, aligning with findings observed in glioblastoma [63]. In addition, MEK1/2 inhibitors showed resistance due to stromal cells in melanoma [64], which agrees with our findings. Importantly, our data revealed that the response to clinically relevant drugs was also affected. Interestingly, Paclitaxel response increased upon co‐culture, corroborating another study using an OC cell line. However, here, Pegylated recombinant human hyaluronidase (PEGPH20) was used to break down the TME for better drug delivery [65]. In addition, we observed resistance to the front‐line treatments Carboplatin and Olaparib. Interestingly, another co‐culture study reported similar findings and showed that OC cells resistance to Carboplatin may be due to induced stemness upon co‐culture [56]. Resistance to Olaparib has been attributed to increased CAFs activation upon treatment [66]. Hence, drug resistance can be altered by various mechanisms: cell–cell/matrix interactions, survival signal secretion from CAFs, or other TME factors stimulating cell survival [67]. However, we identified drugs, Vorinostat and Birinapant, with increased cancer cell killing in co‐culture. Vorinostat, an HDAC inhibitor, disrupts the cell cycle, proliferation, and apoptosis by altering chromatin condensation [68]. Approved for lymphoma [69], Vorinostat has shown poorer results in solid tumors as monotherapy. We tested the Vorinostat and Carboplatin combination, observing synergy in two out of eight *ex vivo* patient samples. This aligns with a clinical study in recurrent platinum‐sensitive OC, where the combination of Paclitaxel, Carboplatin, and Vorinostat showed a 60% objective response rate, long medial overall survival, and good tolerability [70]. Additionally, although only OVCAR8 cells showed increased cancer cell death to Birinapant in co‐cultures, synergy of Birinapant and Carboplatin was observed in three out of eight tested *ex vivo* patient samples. Our results agree with a recent study by Singh et al, where authors used the same drug combination on OC cell lines, patient cells, and xenografts [71]. While drug synergy scoring remains an important parameter for evaluating combination treatment effects, synergy is not essential for clinical benefit, as observed additive effects may enhance treatment response [72, 73]. All patients whose material was used in this study had HGSOC disease at stage III; there was a considerable variability of CA‐125 levels. CA‐125 is used as a biomarker for OC [74] with high pre‐treatment levels of CA‐125 associated with poor survival rates [75]. Our findings suggest that CA‐125 levels may serve as a biomarker to identify a subset of patients more likely to benefit from the combination of Birinapant/Vorinostat and Carboplatin. In addition, samples with high CA‐125 levels exhibit a greater proportion of EMT‐like cells, which may have contributed to cell killing observed with drug combinations. Notably, previous studies have linked HDAC inhibitors to metastasis suppression through EMT inhibition in colorectal cancer [76], suggesting a possible mechanism underlying the observed treatment response. However, further studies are needed to understand the role of the TME in drug response and to validate CA‐125 as a potential biomarker for the patient group that would benefit from combination treatment with Birinapant/Vorinostat.

## Conclusions

In summary, our study highlights the significant role of fibroblasts within the TME, altering cancer cell proliferation, morphology, and cytokine secretion. While cytokine secretion alone could not fully explain the changes observed in co‐cultures, it added to our understanding of the complex cell communication network. Most importantly, our drug screen revealed multiple drugs and drug classes whose efficacy was affected by co‐culture conditions, demonstrating the substantial impact of fibroblasts on cancer cell resistance to the treatment. Importantly, we also discovered drugs that target novel cancer cell phenotypes induced by fibroblasts. In particular, we have identified Vorinostat or Birinapant in combination with Carboplatin as a promising future therapy to improve OC patient outcomes.

## Conflict of interest

OK is a board member and co‐founder for Sartar, advisor to the Knut and Alice Wallenberg Foundation, Novo Nordisk Foundation, and Sitra.

## Author contributions

BSL, PÖ, and GG designed the project. BSL and OK supervised the project. GG and LL conducted the experiments. JL and RB performed targeted DNA sequencing and bioinformatics analysis of this data. GG and OS performed image and/or data analysis. GG and BSL wrote the first draft of the manuscript. UJ, JF, and EM identified patients, obtained patient consent, and provided surgical samples. All authors have read and approved the final manuscript draft.

## Peer review

The peer review history for this article is available at https://www.webofscience.com/api/gateway/wos/peer‐review/10.1002/1878‐0261.70051.

## Supporting information


**Fig. S1.** Cancer cell and fibroblast interactions alter cancer cell proliferation and morphology.

**Fig. S2.** Cytokine profiling reveals differences between monocultures and co‐cultures.

**Fig. S3.** Co‐culture screening reveals cancer cell resistance to drugs in presence of fibroblasts.

**Fig. S4.** Vorinostat and Birinapant in combination with Carboplatin show synergistic effects in cell co‐cultures and *ex vivo* spheroids.

Supplementary Tables.

## Data Availability

Cytokine secretion data (as NPX values) and drug screening results (as DSS, ZIP score, or viability) are available in supplemental material. The following publicly available datasets representing ovarian cancer cell line gene expression, CNV, and mutations were downloaded from the DepMap portal (https://depmap.org/portal) as detailed in the Methods section.
