# Supplementary material for: Systematic profiling of cancer‐fibroblast interactions reveals drug combinations in ovarian cancer
Source: Mol Oncol. 2025 May 24;19(9):2574–93. doi: 10.1002/1878-0261.70051 (PMC12420376; doi:10.1002/1878-0261.70051)
Supplement: Supplementary file 1 — Fig. S1. Cancer cell and fibroblast interactions alter cancer cell proliferation and morphology. [file MOL2-19-2574-s003.pdf]

Supplementaty Figure 1

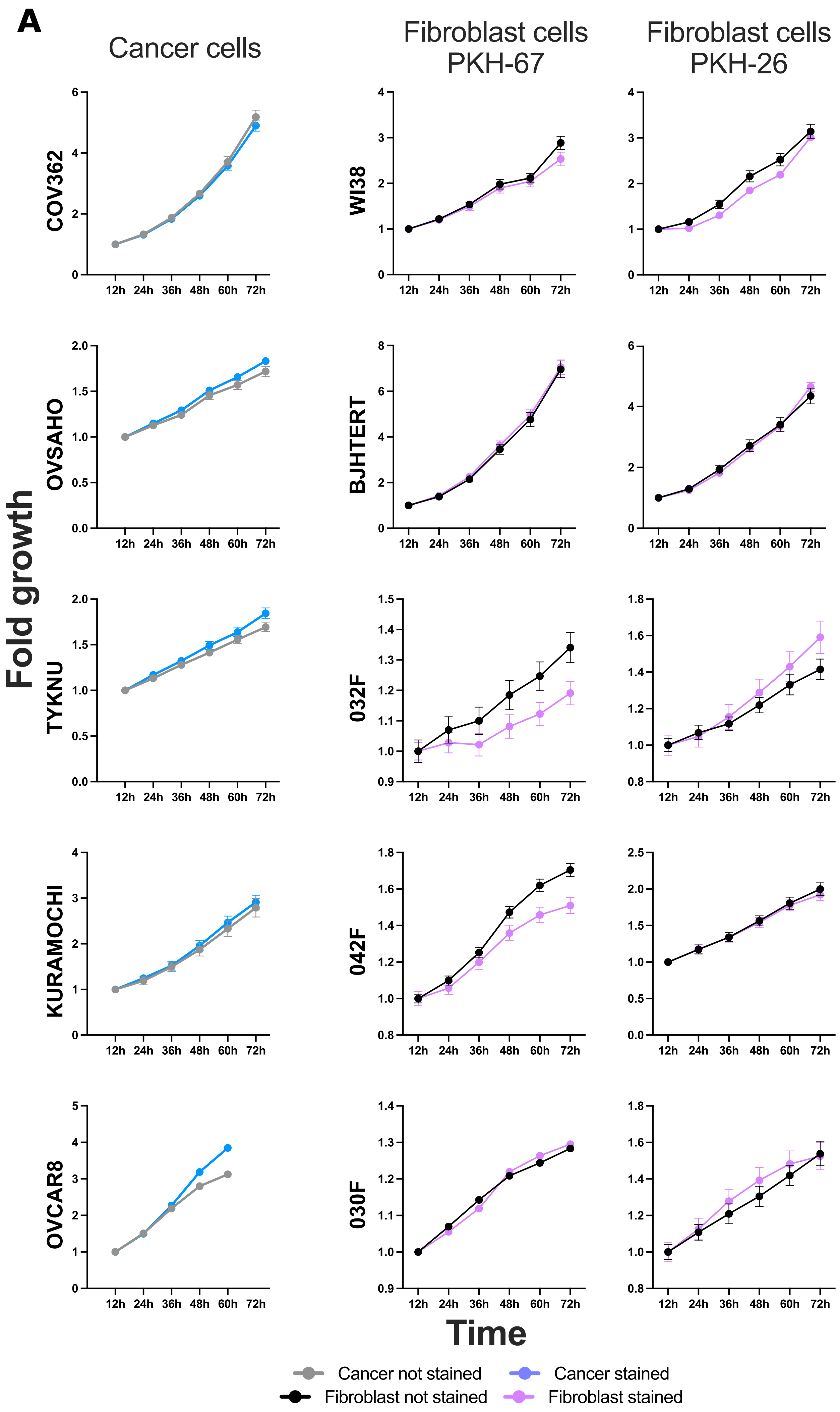

Supplementary Figure 1

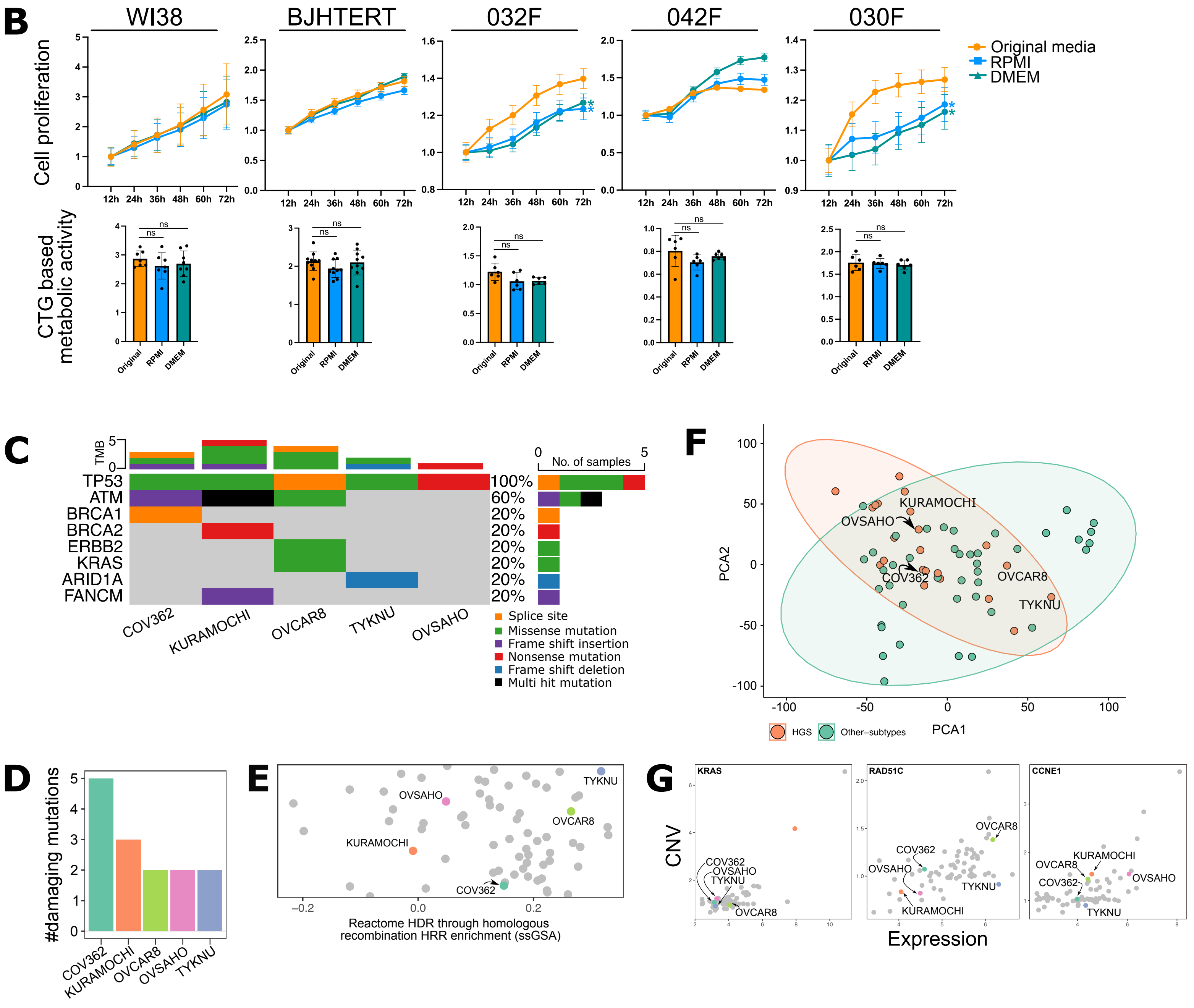

Supplementary Figure 1

H

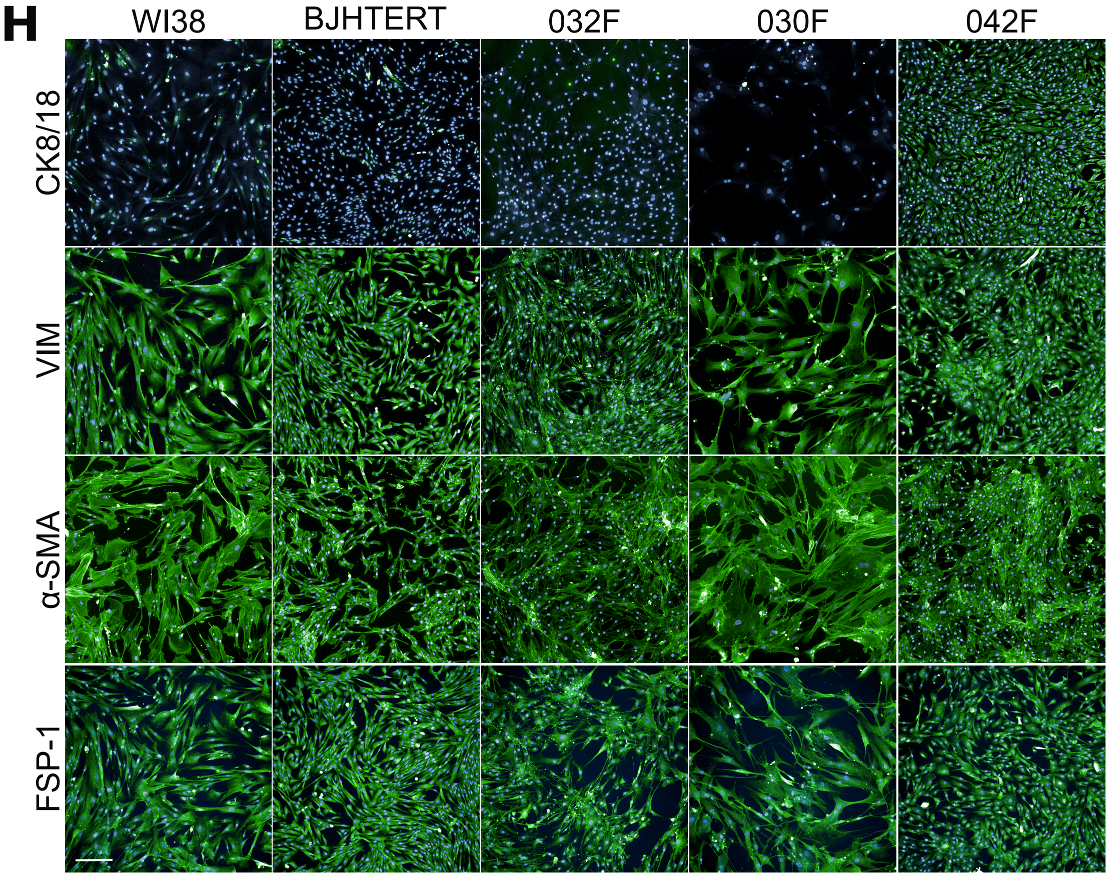

I

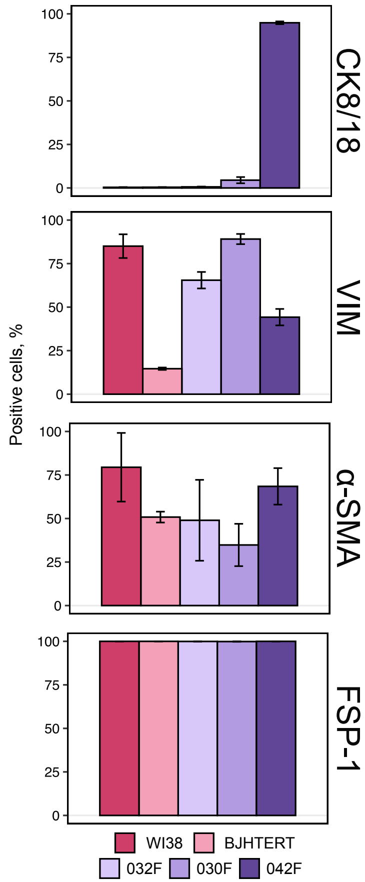

J

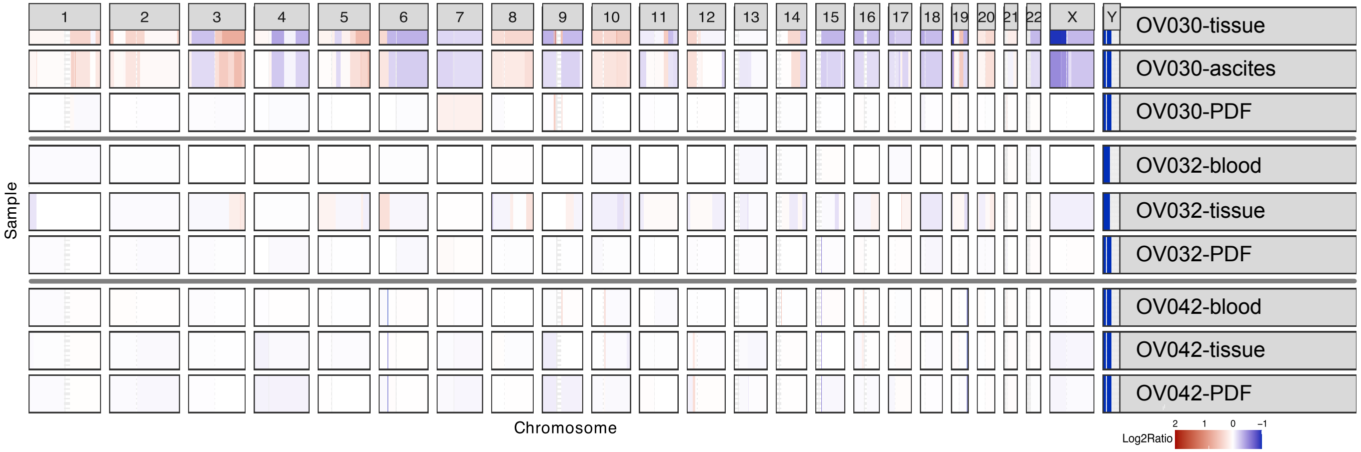

Supplementary Figure 1

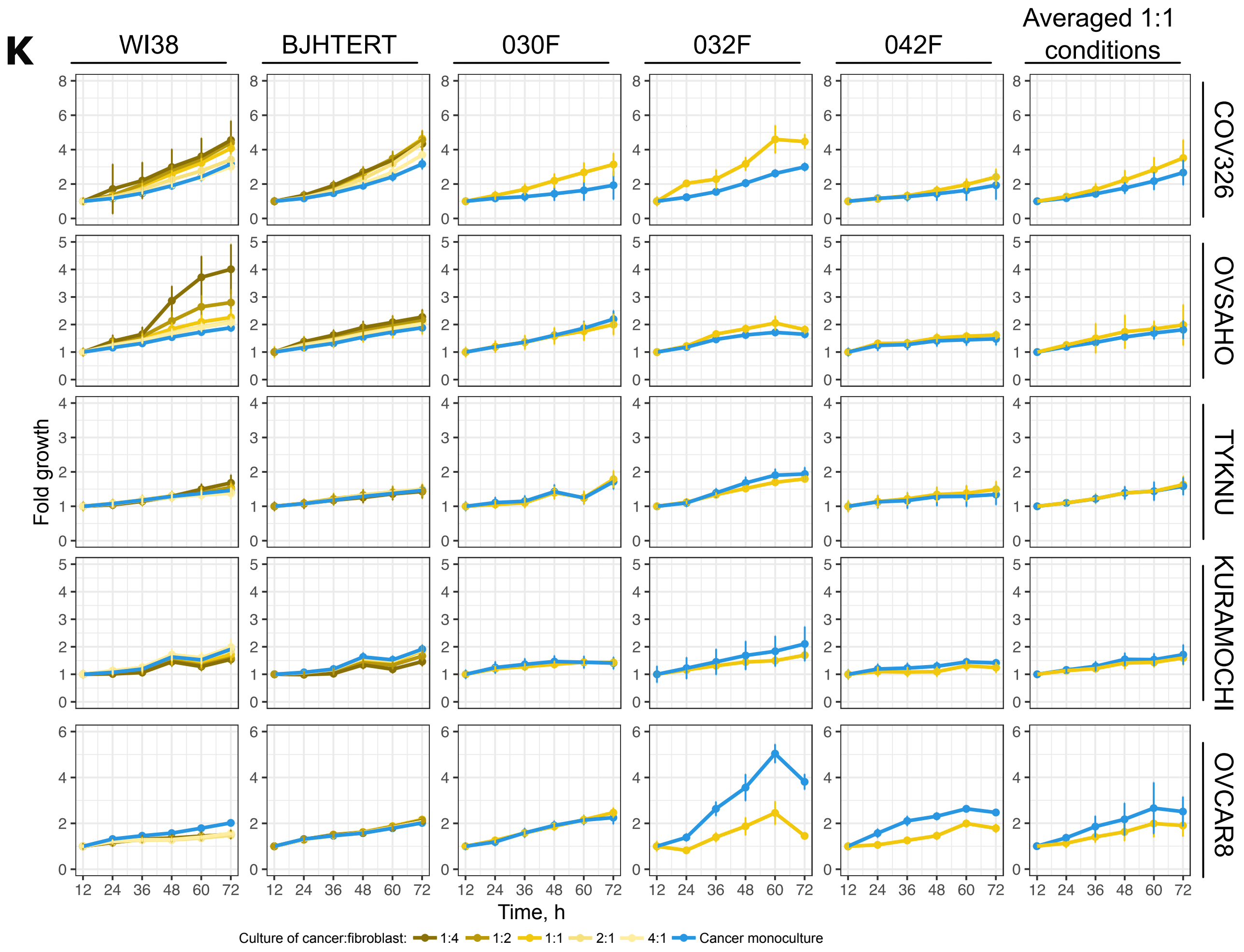

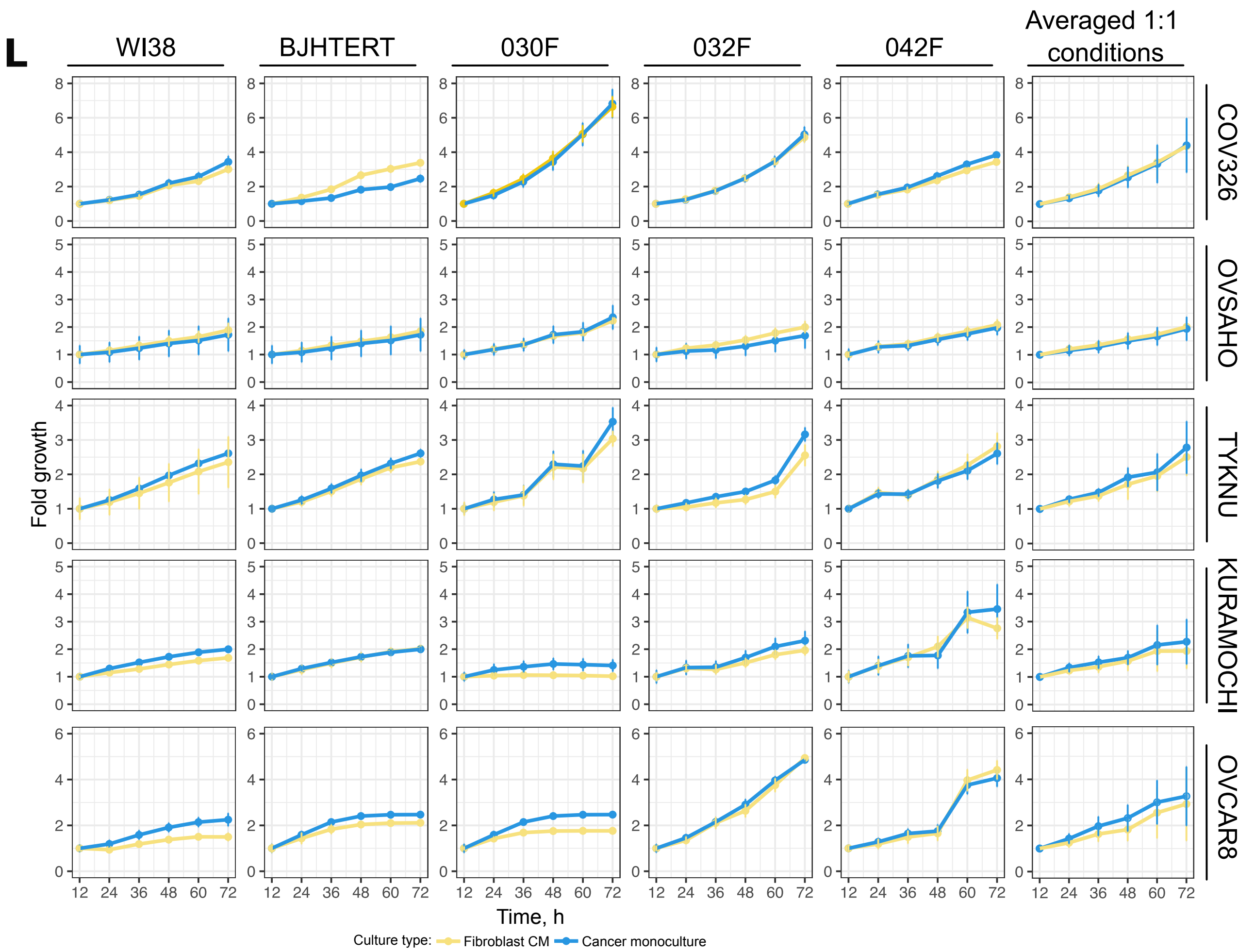

Supplementary Figure 1

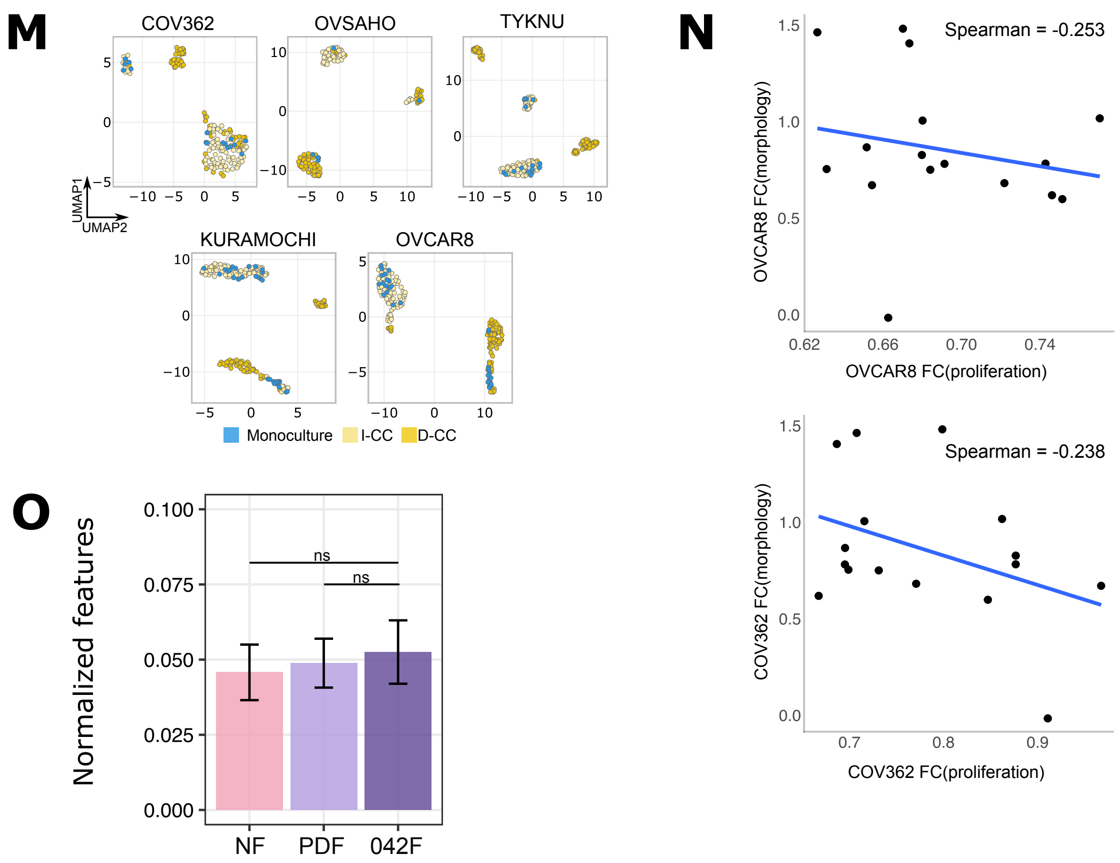

Supplementary Figure 1.

A – To evaluate that if the live-cell staining significantly alters cell proliferation we quantified cancer cell and fibroblast growth over time with and without live cell dyes, error bars indicate ± SD (n=8-16 replicates for each condition).

B – Evaluation of fibroblast proliferation and metabolic activity in three different media used for co-culture assays. The top row depicts fold growth change over time, bottom row shows a bar plots comparing CTG-based metabolic activity fold growth between 0 and 72 h. Original media indicates media normally used for each of fibroblast culture (see methods section for further details), RPMI and DMEM are the medias used in co-culture assays. Error bars in the plots indicate ± SD (n=8 for each condition), $^{*}p<0.05$ using one-way ANOVA.

C – Oncoplot depicting the most common high-grade serous ovarian cancer (OC) genetic aberrations in the five cancer cell lines used in the study. Data extracted from the DepMap portal.

D – Number of damaging mutations in homologous recombination deficiency related genes across the five cancer cell lines used in the study. Visualization is based on data from the DepMap portal. For a list for genes see Sup. Table 6

E – Single sample gene set enrichment analysis (ssGSEA) pathway enrichment of all cell lines annotated as OC in the DepMap portal. Pathway enrichment was computed using molecular signatures for “Reactome HDR through homologous recombination HRR enrichment”. The cell lines used in this study are demarcated in color and with annotations.

F – PCA plot visualizing the variance in gene expression profiles of the OC cell lines available in the DepMap portal with the cell lines selected for this study demarcated. Cell lines annotated as high-grade serous OC (HGS-OC) are shown in orange all other subtypes are light green.

G – Copy number variation (CNV) and gene expression comparison for KRAS, RAD51C, CCNE1 genes in OC cell lines, based on DepMap portal data. Cell lines selected for this study are demarcated.

H– Representative immunofluorescent images of the fibroblast models used in this study for each of the cancer-associated fibroblast-related markers examined, scale bar 200 µm. Green indicates the respective marker fluorescence; blue is nuclear stain).

I– Bar plots representing the percentage of cells expressing tested markers in the fibroblast cells cultures. Each bar shows the mean ± SD (n=6 for each condition).

J – CNV profile of original patient tumor/blood/ascites tissue and the patient derived fibroblasts (PDFs) models used in this study.

K – Cancer cell growth shown as fold change in monoculture and direct co-culture (D-CC) over 72h period, shown as mean ± SD (n=8 for each condition).

L – Cancer cell growth shown as fold change in monoculture and indirect co-culture (I-CC) over 72h period, shown as mean ± SD (n=8 for each condition).

M – UMAP representation of the distribution of morphological features upon different culture conditions, each plot provides a closer look to each cancer cell line cell morphology distribution based on culture conditions, each cell culture condition is represented by the color scheme found in the figure legend.

O – Comparison of normalized morphological features between fibroblast groups, data presented as mean of ± SD (n=4-8 for each condition).

N – Scatterplots representing the correlation between morphological features and growth changes for OVCAR8 and COV362 cell lines.
