# Supplementary material for: Systematic profiling of cancer‐fibroblast interactions reveals drug combinations in ovarian cancer
Source: Mol Oncol. 2025 May 24;19(9):2574–93. doi: 10.1002/1878-0261.70051 (PMC12420376; doi:10.1002/1878-0261.70051)
Supplement: Supplementary file 2 — Fig. S2. Cytokine profiling reveals differences between monocultures and co‐cultures. [file MOL2-19-2574-s005.pdf]

Supplementary Figure 2

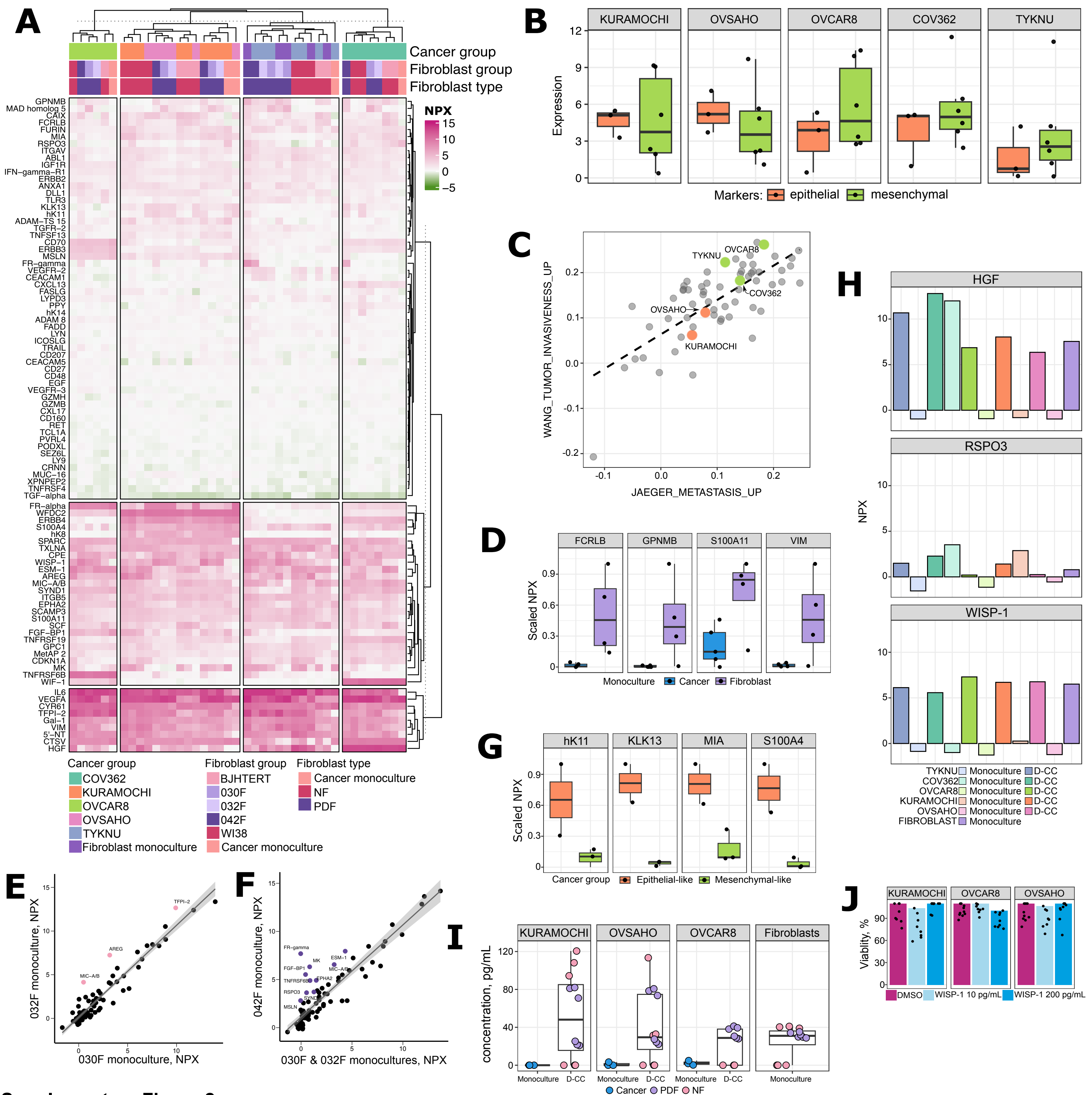

Supplementary Figure 2.

A – Cytokine secretion profiles represented in a heatmap showing normalized protein expression (NPX) values from the Olink panel analysis. Unsupervised sample and cytokine hierarchical clustering performed using the Euclidean distance metric. Legends are shown in the figure for cell types and groups.

B – epithelial-mesenchymal transition related gene expression comparison for each of the cancer cell lines used in this study. DepMap portal gene expression data was used to perform the comparison. For a list for genes see Sup. Table 6.

C – ovarian cancer (OC) cell line gene set enrichment comparison between two pathways using data from DepMap portal. Green dots represent mesenchymal-like cancer cell lines used in this study, orange – epithelial-like cells. The genesets for the single sample gene set enrichment analysis (ssGSEA) are found in the axis titles.

D – Differentially secreted cytokines between cancer and fibroblast monocultures, shown as scaled NPX (0 to 1).

E – Cytokine secretion comparison between 030F and 032F.

F – Cytokine secretion comparison between 042F and remaining two PDFs.

G – Differentially secreted cytokines between epithelial/mesenchymal-like cancer cell monocultures, shown in in scaled NPX values (0 to 1).

H – The most altered cytokine secretion comparison representing cytokine levels in monocultures and co-cultures, shown as NPX values.

I – Concentration of WISP-1 secreted by monocultures and co-cultures, validated with ELISA assay for an independent experiment.

J – Cell viability with and without WISP-1 supplementation in the media.
