# Supplementary material for: Systematic profiling of cancer‐fibroblast interactions reveals drug combinations in ovarian cancer
Source: Mol Oncol. 2025 May 24;19(9):2574–93. doi: 10.1002/1878-0261.70051 (PMC12420376; doi:10.1002/1878-0261.70051)
Supplement: Supplementary file 3 — Fig. S3. Co‐culture screening reveals cancer cell resistance to drugs in presence of fibroblasts. [file MOL2-19-2574-s002.pdf]

Supplementary Figure 3

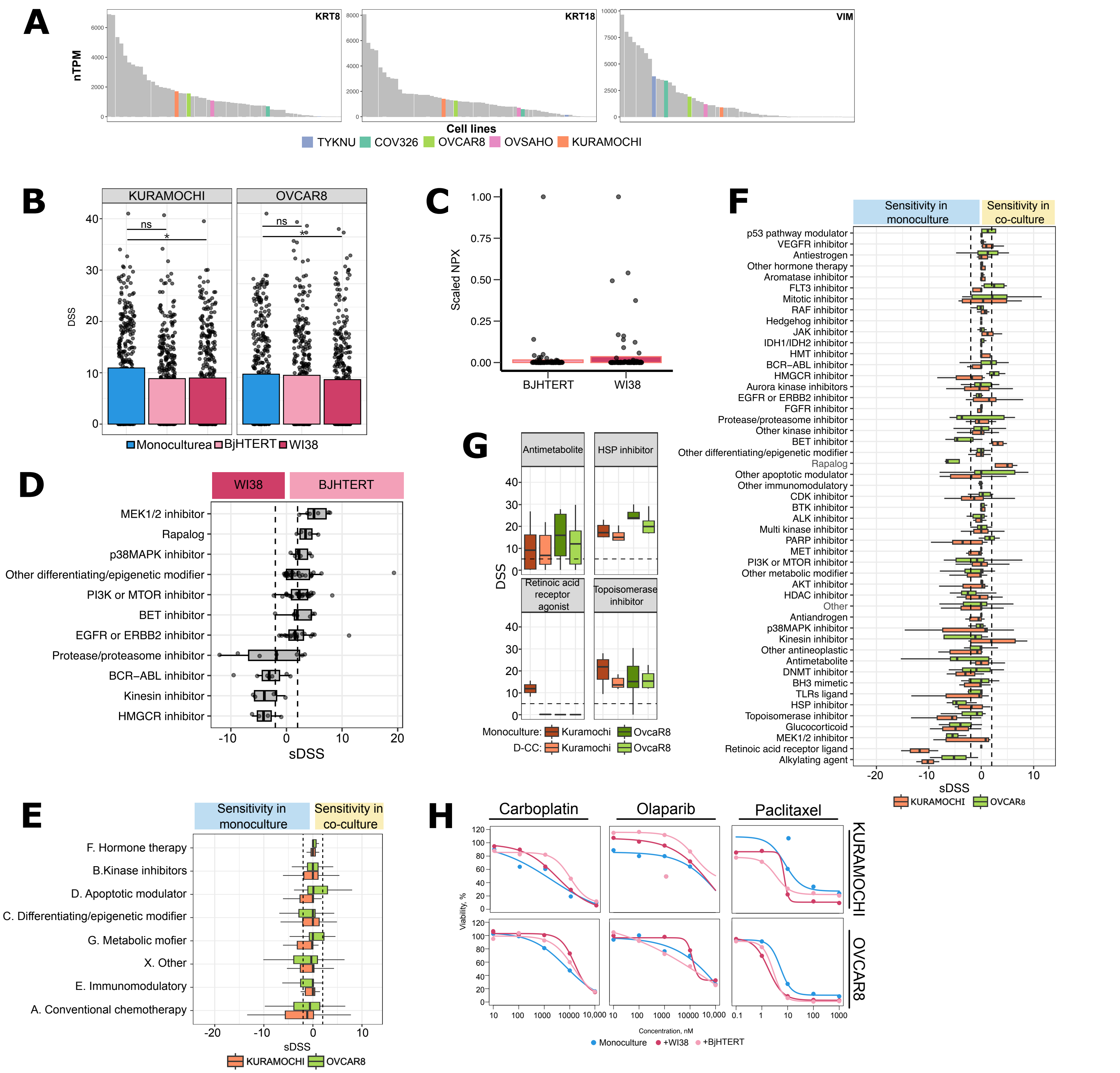

Supplementary Figure 3.

A – Normalized transcripts per million (nTPM) showing keratin-8 (KRT8), keratin-18 (KRT18) and vimentin (VIM) expression levels in ovarian cancer cell lines, where different colors indicate cell lines used in this study. Data retrieved from Human Protein atlas v24.0.

B – Comparison of drug response between cancer monocultures and co-cultures with BJHTERT and WI38 fibroblasts, *p<0.05 from Man-U-Whitney t-test.

C – Normalized cytokine secretion comparison between BJHTERT and WI38, shown as normalized protein expression (NPX) values.

D – Drug subclasses differentially sensitizing cancer cells in co-culture with either BJHTERT or WI38, shown averaged for co-cultures of both cancer cell lines.

E – Overview of cancer KURAMOCHI and OVCAR8 cancer cell drug response to all drug classes in the drug library, shown as selective drug sensitivity score (sDSS), compared to respective monoculture.

F – Overview of cancer KURAMOCHI and OVCAR8 cancer cell drug response to all drug subclasses in the drug library, shown as sDSS, compared to respective monoculture.

G – Additional drug subclasses showing differential effects between cancer cell lines and culture conditions.

H – Drug response curves representing KURAMOCHI and OVCAR8 monoculture and co-culture drug response to standard-of-care treatment.
