# Supplementary material for: Systematic profiling of cancer‐fibroblast interactions reveals drug combinations in ovarian cancer
Source: Mol Oncol. 2025 May 24;19(9):2574–93. doi: 10.1002/1878-0261.70051 (PMC12420376; doi:10.1002/1878-0261.70051)
Supplement: Supplementary file 4 — Fig. S4. Vorinostat and Birinapant in combination with Carboplatin show synergistic effects in cell co‐cultures and ex vivo spheroids. [file MOL2-19-2574-s001.pdf]

Supplementary Figure 4

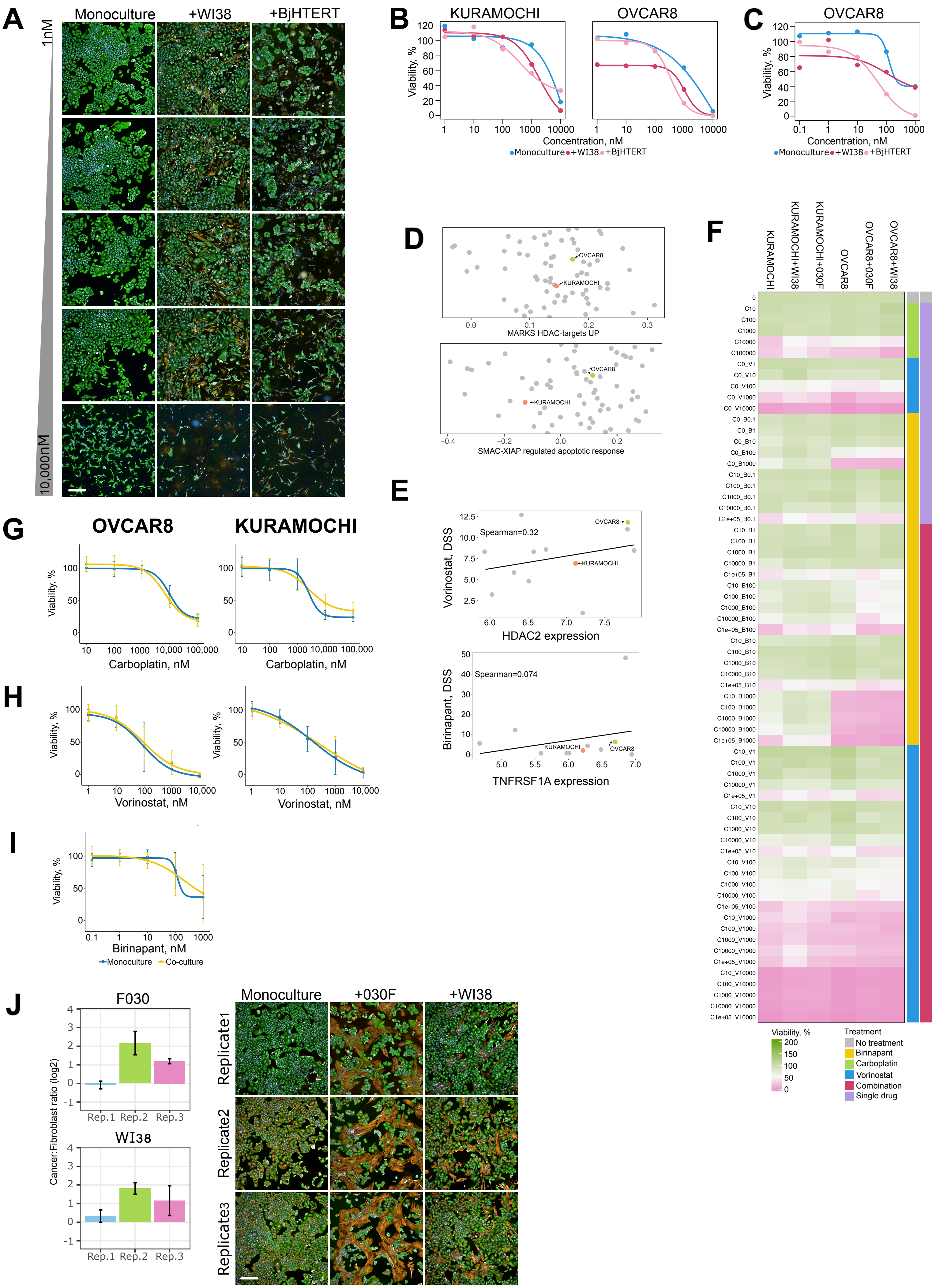

Supplementary Figure 4

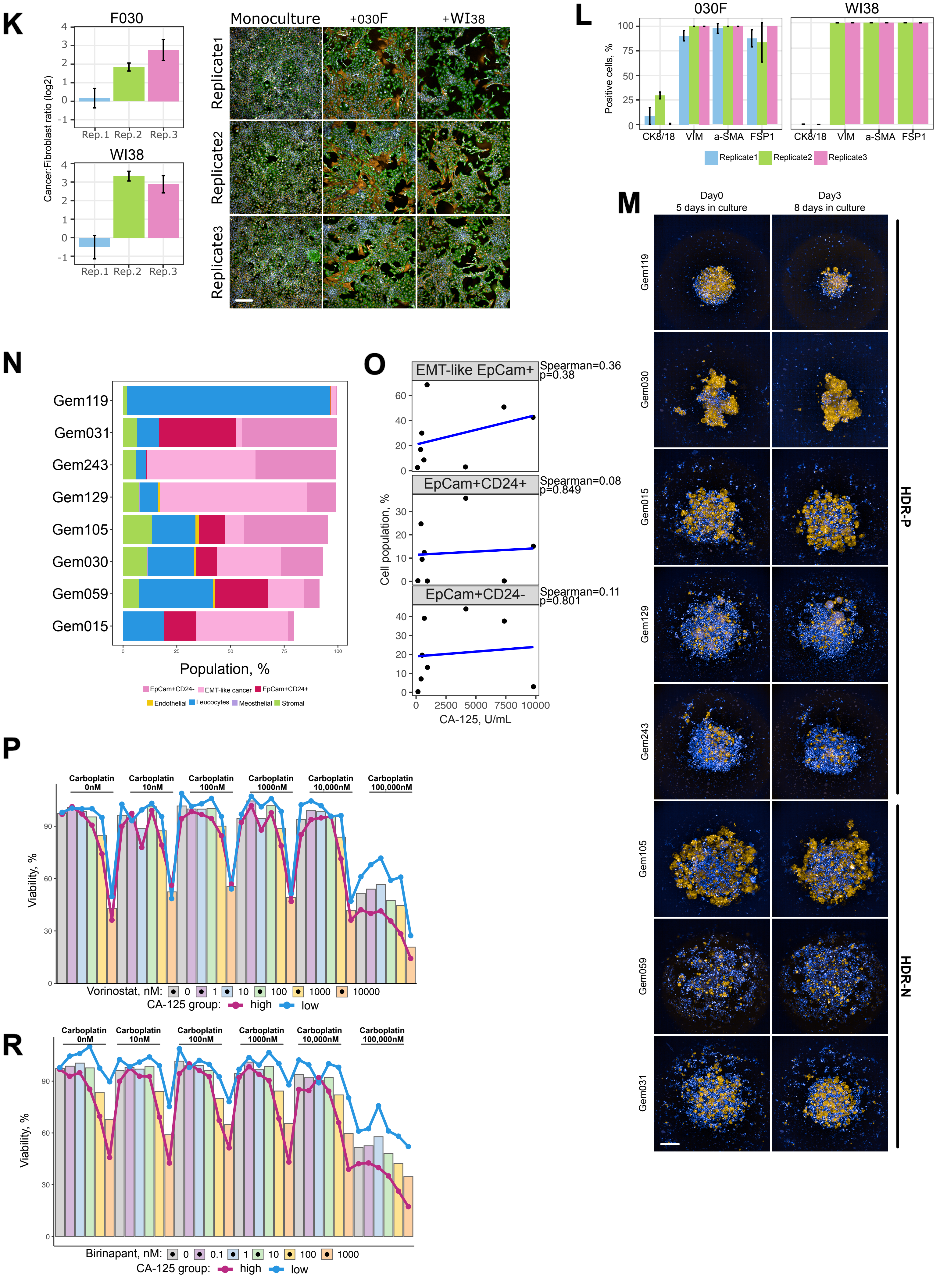

Supplementary Figure 4.

A – IF images, representing KURAMOCHI cell response to 5-fold Vorinostat concentrations after 72 h treatment. Green in the images represent cancer cells, stained with CK8/18, and orange – fibroblast stained with vimentin, blue – nuclei stained with Hoechst, scale bar 200 μm.

B – Drug response curves of KURAMOCHI and OVCAR8 monocultures and co-cultures after treatment with Vorinostat, representing results from high-throughput screen.

C – Drug response curves of OVCAR8 monocultures and co-cultures after treatment with Birinapant, representing results from high-throughput screen.

D – OC cell line distribution for gene enrichment for HDAC-targets upregulation and SMAC-XIAP regulated apoptosis response, based on DepMap portal data.

E – The correlation between Vorinostat and Birinapant drug response and HDAC2 and TNFRSF1A gene expression respectively. Drug response data was generated internally and provided in Sup. Table 15, gene expression data is retrieved from DepMap portal.

F – An overview of the cell viability change presented in a heatmap after 72 h treatment with Vorinostat, Birinapant and Carboplatin. Cancer cells and their cultures are listed in y-axis, drugs and their combinations listed in x-axis, where V – Vorinostat, B – Birinapant, C – Carboplatin and numbers indicate combination of each drug at nM concentration.

Validation data representing drug response curves of KURAMOCHI and OVCAR8 after treatment with G – Carboplatin, H – Vorinostat, I – Birinapant, error bars represent ± SD (n=9).

J, K – Cancer and fibroblast cell ratio comparison between biological replicates, for KURAMOCHI and OVCAR8 respectively. Bar plots represent the ratio between cell types shown in log2 scale, where values >0 represent higher ratio of cancer cells in culture and values <0 indicate higher ratio of fibroblast cells. Images on the right-hand side show cell cultures from control wells without drug treatment, these are representative images showcasing all three replicates with 12 technical replicates each. Images for Replicate2 are duplicates of the "no drug" condition from Fig. 4E shown here to present all three replicates in one panel. Scale bar in the images is 200 μm.

L – CAF marker expression in 030F and WI38 used in combination drug screening at different biological replicates, represented as mean ± SD.

M – Images representing all ex vivo cultures in control wells (treated with DMSO) 5 and 8 days in culture, here yellow represents viable cells, blue – dead cells, scale bar 200 μm.

N – Cell population distribution in samples based on flow cytometry characterization.

O – Scatterplots representing the correlation between cancer cell populations and CA-125 levels in tested samples.

P, R – Cell viability when treated with Carboplatin, Vorinostat and Birinapant single drug and combinations, showing the change of cell viability in CA-125 high and low patient groups.
